# Tau Stabilizes Chromatin Compaction

**DOI:** 10.3389/fcell.2021.740550

**Published:** 2021-10-14

**Authors:** Thomas Rico, Melissa Gilles, Alban Chauderlier, Thomas Comptdaer, Romain Magnez, Maggy Chwastyniak, Herve Drobecq, Florence Pinet, Xavier Thuru, Luc Buée, Marie-Christine Galas, Bruno Lefebvre

**Affiliations:** ^1^Univ. Lille, INSERM, CHU-Lille, Lille Neuroscience and Cognition, UMR-S1172, Alzheimer and Tauopathies, Lille, France; ^2^Univ. Lille, CNRS, INSERM, CHU Lille, UMR 9020, UMR 1277, Canther, Platform of Integrative Chemical Biology, Cancer Heterogeneity, Plasticity and Resistance to Therapies, Lille, France; ^3^Univ. Lille, INSERM, CHU Lille, Institut Pasteur de Lille, U1167 – RID-AGE – Facteurs de Risque et Déterminants Moléculaires des Maladies Liées Au Vieillissement, Lille, France; ^4^Univ. Lille, CNRS UMR 9017, INSERM U1019, CHRU Lille, Institut Pasteur de Lille, Center for Infection and Immunity of Lille, Lille, France

**Keywords:** Tau protein (Tau), chromatin remodeling, chromatin regulation, histone modification and chromatin structure, histone deacetylase inhibitor (HDAC inhibitor), histone (de)acetylation, cancer biology

## Abstract

An extensive body of literature suggested a possible role of the microtubule-associated protein Tau in chromatin functions and/or organization in neuronal, non-neuronal, and cancer cells. How Tau functions in these processes remains elusive. Here we report that Tau expression in breast cancer cell lines causes resistance to the anti-cancer effects of histone deacetylase inhibitors, by preventing histone deacetylase inhibitor-inducible gene expression and remodeling of chromatin structure. We identify Tau as a protein recognizing and binding to core histone when H3 and H4 are devoid of any post-translational modifications or acetylated H4 that increases the Tau’s affinity. Consistent with chromatin structure alterations in neurons found in frontotemporal lobar degeneration, Tau mutations did not prevent histone deacetylase-inhibitor-induced higher chromatin structure remodeling by suppressing Tau binding to histones. In addition, we demonstrate that the interaction between Tau and histones prevents further histone H3 post-translational modifications induced by histone deacetylase-inhibitor treatment by maintaining a more compact chromatin structure. Altogether, these results highlight a new cellular role for Tau as a chromatin reader, which opens new therapeutic avenues to exploit Tau biology in neuronal and cancer cells.

## Introduction

Tau was first described as a neuronal microtubule-associated protein (MAP), regulating microtubule assembly and axonal transport. In the brain, the microtubule associated protein tau (MAPT) constitutes a family of six isoforms containing three or four microtubule binding domains (named 3R and 4R, respectively). The 4R isoforms have a higher affinity for microtubules (Wang and Mandelkow, 2016). In mouse, Tau3R(s) are expressed mostly during development whereas Tau4R becomes the predominant isoform in adult brain. It is thought that the lower affinity of Tau3R for microtubules allows the morphological changes necessary for neuronal differentiation and migration (Lu and Kosik, 2001; Avila et al., 2004; Sergeant et al., 2005). The affinity of Tau for microtubules is also tightly regulated by post-translational modifications. In tauopathies, there is abnormal Tau phosphorylation, leading it to detach from microtubules and favoring aggregation. In addition, several mutations in the MAPT gene have been identified in the inherited frontotemporal dementia and parkinsonism linked to chromosome 17 (FTDP-MAPT). Known mutations either reduce Tau affinity for microtubules or change the ratio (3R/4R) (Goedert and Spillantini, 2000).

Since its original discovery as a brain disease gene, Tau expression has been detected in several non-neuronal cells like kidney, liver, and muscle. Furthermore, Tau is overexpressed in different human breast, gastric, prostate cancer cell lines, and tissues (Gargini et al., 2019). Previous studies have suggested that Tau expression could be a predictive marker for paclitaxel resistance in different cancer types (Wagner et al., 2005; Lei et al., 2020). At the molecular level, it has been demonstrated that Tau protects microtubule from paclitaxel binding by binding to Tubulin (Smoter et al., 2011).

However, recent studies show that the role of Tau is not limited to microtubule dynamics. It has been demonstrated that Tau interferes with several biological processes such as signaling pathways, synaptic functions, RNA metabolism, and DNA integrity and it even contributes to the inflammatory response (Bou Samra et al., 2017; Lebouvier et al., 2017; Sotiropoulos et al., 2017). Most intriguingly, chromatin abnormalities were detected in neurons from several tauopathy models, as well as in human pathological brains. Indeed, there are large-scale changes in histone acetylation, throughout the epigenome in Tau pathologies (Klein et al., 2019). Supporting the likely importance of Tau’s effect on chromatin, overexpression of a FTDP-MAPT mutant in drosophila led to global chromatin relaxation, showed by the loss of H3K9me2 and altered distribution of heterochromatin-associated protein HP1α (Frost et al., 2014). In addition, peripheral cells from patients carrying Tau mutations also displayed chromosome numerical and structural aberrations as well as chromatin anomalies (Payao et al., 1998; Rossi et al., 2018). Frost et al. (2014) further demonstrated that this heterochromatin relaxation, thought to be a consequence of DNA damage induced by oxidative stress, allowed the expression of genes normally repressed in heterochromatin. Among them, dysregulation of transposable elements expression has been shown to be essential in mediating neuronal cell death (Sun et al., 2018). Although the heterochromatin maintenance has been attributed to nuclear Tau in this context, the molecular mechanisms remain elusive (Mansuroglu et al., 2016). Indeed, nuclear Tau has been detected in several cell types (Sjoberg et al., 2006; Mansuroglu et al., 2016; Bou Samra et al., 2017).

In cells, DNA is packaged within the eukaryotic nucleus by association with histones and organized into chromatin domains termed euchromatin and heterochromatin. Euchromatin is associated typically with transcriptionally active regions while heterochromatin with gene silencing. Histone function is modulated by multiple posttranslational modifications, such as methylation and acetylation, and regulate the balance between the two types of chromatin domain. Of such modifications, histone acetylation is generally associated with an open chromatin configuration that facilitates gene transcription. Reversible acetylation is controlled by opposite activities of histone acetyltransferase and histone deacetylase (Allis and Jenuwein, 2016). In the past decades, several histone deacetylase-inhibitors have been developed (Li and Seto, 2016). At the molecular level, these compounds lead to accumulation of acetylated histones and non-histone proteins such as transcription factors, tubulin, and heat-shock proteins, selectively altering gene expression (Glaser et al., 2003; Mitsiades et al., 2004). Global changes in chromatin supra-organization due to histone deacetylase-inhibitors can be also observed using different techniques such confocal laser scanning microscopy or enhanced sensitivity of DNA to nucleases (Toth et al., 2004; Gorisch et al., 2005; Marchion et al., 2005; Bustos et al., 2017). In addition, previous studies show that upon inhibition of acetylation, heterochromatin binding proteins reversibly detach and disperse within the nucleus (Taddei et al., 2001; Robbins et al., 2005; Cowell et al., 2011). The loss of binding of HP1s to heterochromatin is also thought to be important in the histone deacetylase-inhibitor mechanism of action as it induces also abnormal mitosis (Morgan and Shilatifard, 2015).

To dissect the role of Tau in controlling chromatin functions and/or organization, we sought to perturb chromatin structure by inhibiting histone deacetylation with the pan-histone deacetylase-inhibitor trichostatin A (TSA). Here we described Tau as a new histone binding protein that stabilized condensed chromatin under histone deacetylase-inhibitor treatment, in part by preventing post-translational modification of histones. Taken together, our results shed new light on the role of Tau on chromatin organization in neuronal and non-neuronal cells.

## Materials and Methods

### Materials and Plasmids

pGEX vectors encoding Tau and Tau-deletion mutants and pGEX-CBP (aa 1202–1848) fused to GST tag were a kind gift from J.C. Lambert (INSERM U1167, Lille, France) and C. Smet-Nocca (UMR8576, Villeneuve d’Ascq, France), respectively. GFP-HP1β was a gift from Tom Misteli (Addgene plasmid # 17651). pGL4.31(luc2P/GAL4 UAS/Hygro) was purchased from Promega. Short hairpin Tau and RNA Ctrl vectors were purchased from Santacruz. pcDNA3-Tau4R has been described elsewhere (Lippens et al., 2004; Chauderlier et al., 2018). GAL4-Tau4R was obtained by inserting an in-frame Tau1N4R (referred to as Tau4R) cDNA isoform into the pM GAL4 DNA-BD cloning vector (Clontech). Purified histones and recombinant histones were obtained from EpiCypher and New England Biolabs, respectively. TSA and BIX 01294 (Sigma) were reconstituted in dimethylsulfoxide and Tetracycline (Sigma) in ethanol.

### Cell Culture and Transfection

SH-SY5Y human neuroblastoma cells expressing Tau4R tagged with the streptavidin-binding peptide (SBP) [referred as SH-SY5Y-(SBP)Tau4R] (Chauderlier et al., 2018), SH-SY5Y-Tet-on-Tau4R, Hela cells, MCF7, and MDA-MB-231 were cultured in Dulbecco’s Modified Eagle’s Medium with 10% fetal bovine serum, 2 mM L-glutamine and 50 U/ml penicillin/streptomycin (Gibco) at 37°C in 5% CO_2_ humidified air. Transient and stable transfections experiments were performed using the lipofectamine 3000 reagent (Invitrogen). Transient luciferase assays were performed with the dual-luciferase assay system (Promega). For stable clones, luciferase activities were measured using the luciferase assay system (Promega) and normalized against protein concentration. To isolate stably transfected clones, Hela cells were transfected with the pGL4.31(luc2P/GAL4 UAS/Hygro) (referred to as the GAL4UAS stable Hela cell line) and selected with hygromycin (200 μg/ml). Of 6 clones tested for reporter activity, one clone was chosen for further studies. MCF7 and MDA-MB-231 cells were transfected with short hairpin Tau or RNA Ctrl vectors and selected with puromycin (1 μg/ml). Clones were isolated and tested for Tau expression.

### *In vitro* Chromatin Assembly

*In vitro* chromatin was obtained using the chromatin assembly kit (Active Motif) according to the manufacturer’s guidelines. Assembled chromatin was then incubated with purified GST or GST-Tau and subjected to limited micrococcal nuclease digestion.

### Micrococcal Nuclease Assays

Nuclei, from SH-SY5Y-Tet-on-Tau4R, induced, or not, with 1 μg/ml tetracycline, 24 h before adding the indicated concentration of trichostatin A (24 h), were resuspended in TM2 (10 mM Tris at pH 7.4, 2 mM MgCl2, and protease inhibitor mixture) then CaCl2 was added to 1 mM and subjected to micrococcal nuclease digestion. The reaction was stopped with addition of 2 mM final EGTA and treated with RNase and then with Proteinase K. The purified DNA was electrophoresed through a 20 cm 1.5% agarose gel and visualized with ethidium bromide. Band intensities were quantified using the ImageJ Gel Analysis program.

### Salt Fractionation of Nucleosomes

Salt fractionation of nucleosomes was performed as described previously (Teves and Henikoff, 2012). Aliquots of each fraction were collected for western-blot analysis or DNA extraction. To analyze histone H3 and H4 post-translational modifications, the supernatants of each fraction was subjected to streptavidin pulldown of Tau fused in frame with the streptavidin binding peptide by incubation with 20 μL of M-280 streptavidin beads (Dynal) in TNE buffer (10 mM Tris–HCl, pH 7.5, 200 mM NaCl, 1 mM EDTA). Bound materials were eluted with biotin (Invitrogen) and analyzed with the EpiQuik^TM^ Histone H3 or H4 Modification Multiplex Assay Kit (EpiGentek) according to the manufacturer’s guidelines.

### RNA Preparation and Real-Time PCR

Total RNA was prepared using RNeasy Mini kit (Qiagen). Reverse transcription (RT) was performed using random hexamers as recommended by the manufacturer (Applied Biosystems). cDNAs were analyzed by PCR amplification using the TaqMan PCR master mix (Applied Biosystems) and a mix of RPLO primers and probes. The different probes were purchased from Applied Biosystems (assay on demand kit). Reactions (40 cycles) and data analysis were carried out with an ABI Prism 7700 (PerkinElmer).

### Chromatin Immunoprecipitations

The ChIP protocol used was previously described (Lefebvre et al., 2006). Primers sequences for the pGL4.31 (luc2P/GAL4 UAS/Hygro) promoter were: forward, 5′-TTCGGAGTACTGTCCTCCG-3′ and reverse, 5′-GGTAGA ATGGCGCTGGGCCC-3′; for p21 promoter: distal forward 5′-TGCTTCCCAGGAACATGCTTG-3′ and reverse 5′-C TGAAAACAGGCAGCCCAAGG-3′; proximal forward 5′-GCAGAGGAGAAAGAAGCCTG-3′ and reverse 5′-GC AGAGGAGAAAGAAGCCTG-3′; p21 (transcription start site) TSS forward: 5′-GCAGAGGAGAAAGAAGCCTG-3′ and reverse 5′-GCTCTCTCACCTCCTCTG3′; and for GAPDH TSS: forward 5′-GGCTCCCACCTTTCTCATCC-3′ and reverse 5′-GGCCATCCACAGTCTTCTGG-3′. Antibodies used in the studies included the following: anti-acetylated H3 (ac-H3) (Active motif, 39139) and anti-Tau1 (Millipore, MAB3420). All ChIP analyses were performed in at least two independent experiments.

### Peptide Binding Assay

A total of 100 ng of purified GST-Tau was incubated with 2 μg of biotin-labeled synthetic peptides corresponding to the N-terminal tail of histone H4 (EpiCypher) in a buffer containing 50 mM Tris–HCl at pH 7.5, 150 mM NaCl, 0.5 mM DTT, and 0.25% Non-idet P-40 for 4 h at 4°C, followed by incubation with 20 μL of M-280 streptavidin beads (Dynal). Peptide sequences were derived from human histone H4, amino acids 1–23 (SGRGKGGKGLGKGGAKRHRKVLR). Beads were washed, and bound material was eluted with 2× sample buffer. Bound materials were resolved on SDS/PAGE and immunoblotted as described below.

### Western-Blot Analysis

Western-blot analysis was carried out using primary antibodies directed against histone H3 (Millipore, 07-690), H4 (Santacruz, sc10810), H2A (Santacruz, sc8648), H2B (Active Motif, 39125), and Tau C-ter (Galas et al., 2006) as described previously (Chauderlier et al., 2018).

### GST-Pulldown Assays

GST-pulldown experiments were performed as described previously using 1 μg of GST or GST-Tau proteins and the equivalent amount of indicated proteins (Chauderlier et al., 2018).

### Immunofluorescence

MCF7shctrl, MCF7shTau, or transfected Hela cells with Tau4R and GFP-HP1β were fixed in 4% paraformaldehyde for 30 min at room temperature. Permeabilization was carried out in 0.2% Triton X-100 in phosphate-buffered saline for 10 min at room temperature. After 1 h saturation in 2% bovine serum albumin, immunostaining was performed using Tau antibody (recognizing the C-terminal domain of Tau). Tau staining was revealed with a goat anti-rabbit IgG antibody coupled to Alexa Fluor^®^ 568 (Molecular Probes). Nuclear staining was performed by adding 1/2000 DAPI in phosphate-buffered saline for 10 min. Slides were then analyzed with a Zeiss LSM710 confocal laser scanning microscope (63× magnification). Images were collected in the Z direction at 0.80 μm intervals and quantifications were realized using the ImageJ plug-in.

### ELISA Measurements

Cellular levels of Tau protein were measured in serial dilution of cell lysates with a home-made tau ELISA kit. MCF7shCtrl and MCF7shTau cells were lysed in 100 μL RIPA (150 mM NaCl, 1% NP40, 0.5% sodium deoxycholate, 0.1% SDS, 50 mM Tris–HCl, pH 8, and completed with protease inhibitors). Then, 96-well microtiter plates (MaxiSorp F8; Nunc, Inc.) were coated overnight at 4°C with 100 ng/well of our home-made antibody 9H12 (recognizing the 162–175 central region of tau) in a carbonate buffer (50 mM NaHCO3, pH 9.6). After five washes with PBS containing 0.05% Tween (PBS-T), saturation was performed using 200 μL of PBS-T with 2% casein (PCT) for 1 h at 37°C. After five washes with the PBS-T buffer, 50 μL of diluted cell lysates in PCT buffer were incubated for a further 1 h at 37°C. The standard containing 1N4R recombinant tau were also incubated. Then, 50 μL of the detection antibodies [home-made TauE13N (7F5)] diluted at 1/1000 in PCT buffer were incubated overnight at room temperature. After five washes with PBS-T, followed a 1/32000 diluted anti-mouse IgG1 secondary antibody incubation. After washes, 100 μl of TMB solution (one pellet of TMB diluted in citrate/phosphate buffer supplemented by 1/5000 H_2_O_2_) was added to each wells and incubated for 30 min. The reaction was stopped by addition of 50 μL of sulfuric acid. Optical density was measured with a spectrophotometer (Multiskan Ascent, Thermo LabSystem) at 450 nm.

### Cell Cycle Analysis, Cell Death, and Apoptosis

Cell cycle, Cell death, and Apoptosis were analyzed with the Click-It Plus EdU Alexa Fluor 647 kit (Thermofisher), Zombie NIR dye (Zombie NIR Fixable Viability Kit – BioLegend), and Annexin V-FITC Apoptosis Detection Kit II (Calbiochem) according to the manufacturer’s guideline. Fluorescence was analyzed using a LSR FORTESSA X20 cytometer (Becton Dickinson).

### Statistical Analysis

Data are mean ± SD. Statistical tests were carried out using GraphPad Prism software (GraphPad Inc.). Statistical significance between groups was analyzed with Wilcoxon–Mann–Whitney test and Mann–Whitney tests. A *p*-value less than 0.05 was considered significant.

## Results

### Tau Knock-Down Increases Breast Cancer Cell Line Sensitivity to Trichostatin A

**FIGURE 1:**
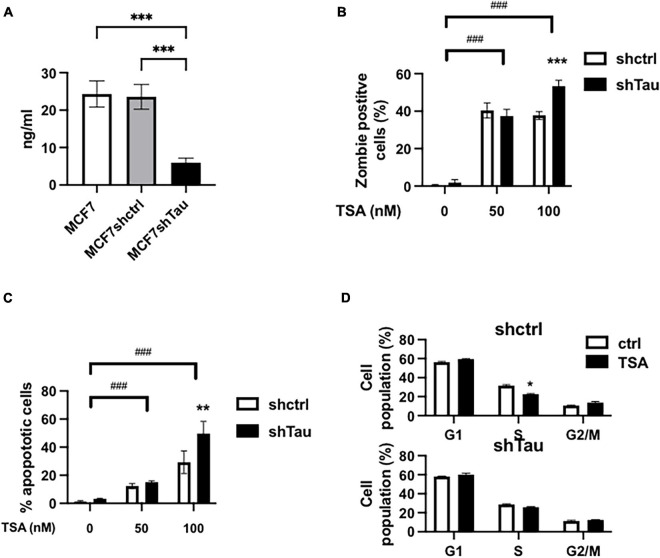
Tau inhibition increases MCF7 breast cancer cell line sensitivity to TSA. **(A)** Tau protein expression in MCF7, MCF7shctrl, and shTau were quantify by ELISA. **(B)** Effect of 50 and 100 nM TSA (48 h) on cell death in the MCF7shctrl and MCF7shTau subclones. Cell death was determined by staining and flow cytometric analysis as described in section “Materials and Methods.” **(C)** Effect of 50 and 100 nM TSA (48 h) on apoptosis in the MCF7shctrl and MCF7shTau subclones. Apoptosis was determined by flow cytometric analysis of the PI-positive and Annexin-V-positive cells as described in section “Materials and Methods.” **(D)** Cell cycle distribution was determined by FACS analysis of combined propidium iodide and EdU staining in MCF7shctrl and MCF7shTau in the absence or presence of 100 nM TSA, 48 h. Data are mean ± SD, *P < 0.05, **P < 0.01, ***P < 0.001, ^###^*P* < 0.001. All results are representative of three independent experiments.

In this study we sought to investigated the role of Tau in controlling chromatin structure and functions. For this we took advantage of breast cancer cell lines that are known to express Tau. Indeed, Tau expression has been extensively characterized in breast cancer cell lines and tumors (Rouzier et al., 2005; Matrone et al., 2010; Spicakova et al., 2010; Li et al., 2013). We choose to overexpress either small hairpin RNA control (shctrl) or targeting Tau (shTau) in MCF7 and MDA-MB-231 cells. We characterized the efficiency of Tau mRNA knock-down in several clones compared to the parental shctrl cell lines. Tau protein level decreased 75% in the selected MCF7shTau clones (Figure 1A and Supplementary Figure 1).

To dissect the role of Tau in controlling chromatin we sought to perturb chromatin structure by inhibiting histone deacetylation with TSA. First, we evaluated cell death, apoptosis and cell cycle progression by flow cytometric analyses in the two cell lines (as above we just illustrate our results with MCF7 cells in the main paper). We tested the effect of TSA (50 and 100 nM) in the presence or absence of short hairpin RNAs (Tau and Control). The percentage of dead cells were minimal in the absence of TSA (0.5 and 1.8% for MCF7shctrl and shTau, respectively) (Figure 1B). After 48 h TSA treatment (50 nM), we observed a increase in the number of dead cells for MCF7shctrl (40.3%) and MCF7shTau (37.4%). Increasing TSA concentration to 100 nM did not modify the proportion of dead cells for MCF7shctrl (37.7%). However, cell viability was further decreased in the absence of Tau (53.4%). We observed a similar response for apoptosis (Figure 1C). At 50 nm TSA, we observed a similar percentage of apoptotic cells for shctrl (12.3%) and shTau cells (17.1%). The percentage of TSA-mediated apoptotic cells was higher at 100 nM for MCF7shctrl cells (29.7%) and was further increased for MCF7shTau (48.2%). We obtained similar results on apoptosis using siRNA targeting sequence different from shRNA and ruling out a possible off-target effect (Supplementary Figure 2).

We next performed cell cycle analyses (Figure 1D). Indeed, treatment with HDACi can result in cell cycle arrest at the G1/S and/or G2/M checkpoints (Newbold et al., 2016). The results revealed that TSA (100 nM) treatment did not impact significantly on cell population at these two checkpoints in MCF7shctrl and shTau cells. This cell cycle analysis thus demonstrated that Tau expression did not impact on TSA-induced cell cycle arrest. Note that for all the above experiments we obtained similar results in MDA-MB-231 cells (Supplementary Figure 3).

Collectively, these results indicated that Tau inhibition increases TSA sensitivity toward apoptosis and cell death in different breast cancer cell lines independently of cell cycle arrest.

### Tau Prevents Trichostatin A-Dependent Chromatin Remodeling

**FIGURE 2:**
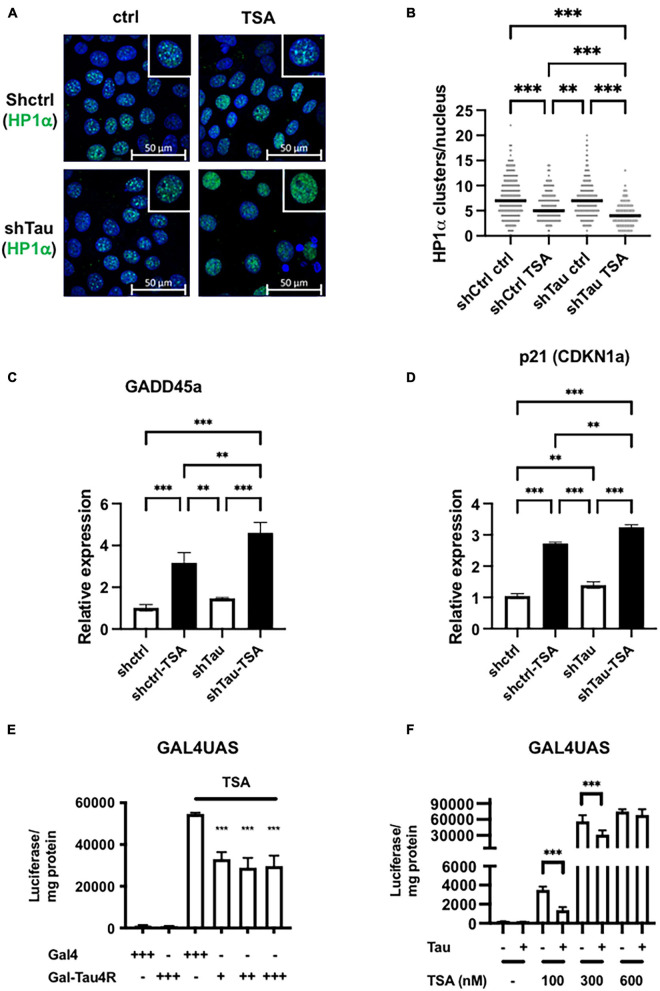
Tau expression prevented TSA-dependent chromatin remodeling. **(A)** Representative confocal sections of MCF7shctrl and shTau cells untreated or treated with 100 nM TSA for 24 h. DNA was revealed by DAPI and HP1α was immuno-localized. **(B)** Quantification of HP1α clusters per nuclei visualized as described previously and realized on three independent experiments. **(C)** GADD45a and **(D)** p21 (CDKN1a) expression in MCF7shctrl and shTau cells untreated or treated with 100 nM TSA for 24 h were analyzed by real-time PCR and normalized to RPLO. Results are expressed, relative to the basal activity set to 1, as the mean ± SD of three independent assays. **(E)** Tau tethering prevents adjacent reporter gene activity induced by TSA. GAL4UAS responsive luciferase reporter Hela stable cell line was transfected with GAL4DBD (GAL4) or GAL4DBD-Tau4R and then, 24 h later, treated with TSA (600 nM) for 24 h. The luciferase activity was determined as described in in section “Materials and Methods.” **(F)** Ectopic Tau4R expression prevents adjacent reporter gene activity induced by TSA. The GAL4UAS responsive luciferase reporter Hela stable cell line was transfected with a plasmid encoding Tau4R for 24 h. Then, cells were treated with an increasing concentration of TSA, as indicated for 24 h. The luciferase activity was determined as described in section “Materials and Methods.” Data are mean ± SD,^∗∗^*P* < 0.01, ^∗∗∗^*P* < 0.001 vs. control.

The above results suggested that Tau modulates the cellular responses to histone deacetylase inhibitors. As histone TSA is known to induce the spreading heterochromatin-associated proteins, we next assessed the effect of TSA on the fate of the endogenous HP1α in shTau-knockdown MCF7 cells (Taddei et al., 2001). Without TSA, MCF7 cells had around eight HP1α clusters per nucleus in both sh-control and Tau-knockdown cells. TSA reduced this to five clusters and even further to just three HP1α clusters per nucleus where Tau was knocked down (Figures 2A,B). Same results were found in other cellular models such as SH-SY5Y-Tet-on-Tau4R. In addition, western-blot analysis revealed that TSA treatment did not induce significant variations in HP1s and H3K9me1/2/3 levels between control and treated cells (Supplementary Figure 4). These data showed that Tau prevented PCH disruption induced by TSA in an independent manner of HP1s and H3K9me2/3 levels.

As well as disrupting pericentromeric heterochromatin, TSA affects histone acetylation at specific promoters and thereby influences chromatin structure and gene expression, of, for example, growth arrest DNA damage gene 45a (GADD45a) and p21 (CIP1/WAF1) (Richon et al., 2000; Hirose et al., 2003). As expected, these two genes were significantly upregulated by TSA in MCF7shctrl cells, as detected by RT-qPCR (3- and 2.5-fold induction, respectively). Importantly this effect was exacerbated in cells with reduced Tau expression (4.6- and 3.2-fold induction, respectively) (Figures 2C,D). This indicates that Tau expression represses in part TSA-induced chromatin remodeling and gene expression.

To directly probe how Tau prevents TSA-induced chromatin remodeling and gene expression, we next used Hela cells as they lack endogenous Tau. First, we generated a stable GAL4UAS (Upstream Activation Sequence)-responsive luciferase reporter Hela clone that we subsequently selected for TSA-induced luciferase activity. Indeed, Gal4 or other tethering systems have been previously used for example to study the effects of HP1 or the insulinator CTCF on chromatin structure (Li et al., 2003; Ahanger et al., 2014). In our preliminary dose/response experiments, highest luciferase activity was observed at 600 nM TSA and we used this concentration in the following experiments. We next examined the resistance of Tau proteins to salt extraction. Indeed, salt solubility has been already used to monitor binding of proteins to the chromatin compartment (Lichota and Grasser, 2001). We found that only Tau4R isoforms but not Tau3R were tightly bound to chromatin and could potentially impact directly on chromatin structure/functions (Supplementary Figure 5). We therefore targeted Tau4R to UAS responsive elements by fusing Tau4R to the heterologous DNA-binding domain GAL4 and transfected this plasmid into Hela stable clone. Importantly we found the luciferase activity was significantly reduced (50%) in the presence of GAL-Tau4R (Figure 2E). This demonstrates that the presence of Tau on the promoter reduced TSA-induced gene expression. We next wondered if this effect was due to the tethering of Tau4R to the promoter or whether this was independent of Tau4R fusion to GAL4. To this end, the wild-type Tau4R (i.e., without the GAL4 fusion) was transfected into a Hela clone and treated with various concentrations of TSA, up to 600 nM. Tau4R overexpression reduced the luciferase activity at 100 and 300 nM by around 50% decreased, whereas at 600 nM TSA there was no reduction (Figure 2F). The fact that the presence of Tau4R decreased the TSA-inducibility of the GAL4UAS responsive luciferase reporter gene suggests that Tau, in particular Tau4R isoforms, modulated the effects of histone acetylation on gene expression.

### Tau4R Regulates Histone Deacetylase Inhibitor Induced Genes Through Direct Binding to Chromatin

**FIGURE 3:**
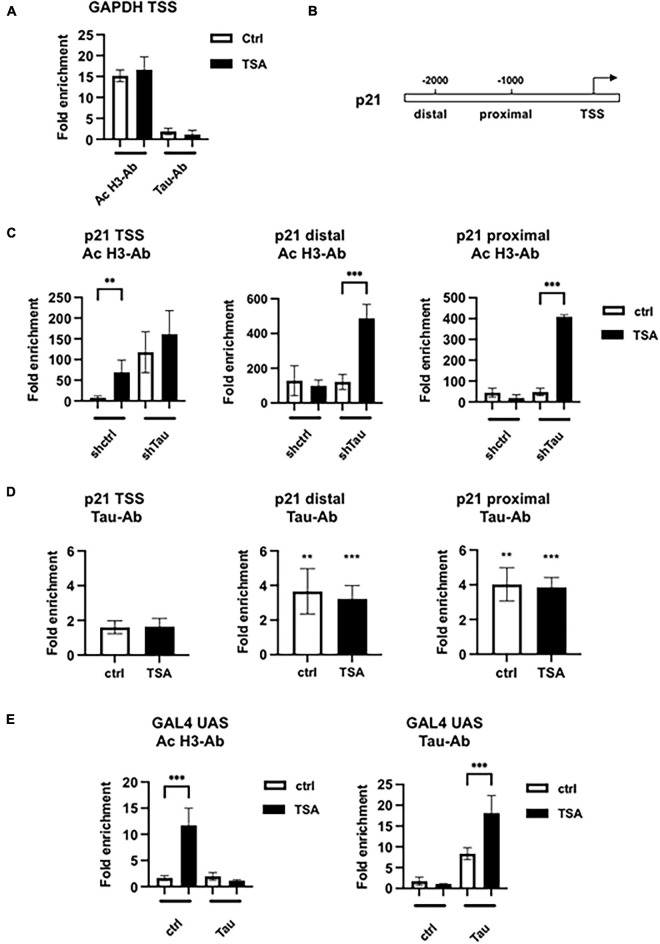
Histone H3 acetylation and Tau occupancy at different promoters. **(A)** Chromatin immunoprecipitations-qPCR analysis of H3 acetylation and Tau occupancy on the GAPDH promoter in MCF7shctrl cells in the indicated conditions. MCF7shctrl cells were subjected to cross-linking by 1% formaldehyde. Chromatin fragments were then immunoprecipitated using antibodies (Ab) against acetylated H3 (ac-H3) or Tau and analyzed by quantitative PCR for the presence of the GAPDH promoter. Quantification of enrichment is represented as fold-enrichment relative to IgG. **(B)** Functional organization of the p21 promoter. **(C)** ChIP-qPCR analysis of H3 acetylation in MCF7shctrl or shTau cells or **(D)** Tau occupancy in MCF7shctrl cells on the p21 promoter. Quantification of enrichment is represented as fold-enrichment relative to IgG. Data are mean ± SD over IgG control, **P < 0.01, ***P < 0.001. **(E)** ChIP-qPCR analysis of H3 acetylation or Tau occupancy on the stably transfected GAL4UAS responsive luciferase reporter transfected, or not, with Tau4R, then 24 h later treated with 100 nM TSA for 24 h. Cells were then subjected to cross-linking by 1% formaldehyde. Chromatin fragments were then immunoprecipitated using antibodies (Ab) against acetylated H3 or Tau and analyzed by quantitative PCR for the presence of the GAL4 UAS promoter. Quantification of enrichment is represented as fold-enrichment relative to IgG. Data are mean ± SD,**P < 0.01, ***P < 0.001.

The above results suggest that Tau4R binds directly to chromatin to regulate gene expression. To test this hypothesis, we next performed chromatin immunoprecipitations assays. Antibodies specific for ac-H3 and Tau were used to immunoprecipitate formaldehyde-cross-linked sonicated chromatin from MCF7 short hairpin Tau knockdowns and control cells treated, or not, with 100 nM TSA. Quantitative PCR analysis of input or immunoprecipitated DNA using ac-H3 and Tau antibodies was carried out to detect different promoter regions. As a control of non-TSA-inducible gene, we first used primers encompassing the transcription start site of the GAPDH promoter. No change in the level of ac-H3, nor Tau binding, were detected after TSA treatment (Figure 3A). We next examined the extent of ac-H3 and Tau occupancy within the p21 promoter associated chromatin, for which activity upon TSA treatment and Tau depletion have been characterized previously (see Figures 2D, 3B). As shown in Figure 3C, TSA induced a strong acetylation in shctrl cells (70- vs. 7-fold enrichment compared to IgG) while a high level of ac-H3 was detected in shTau cells, irrespective of TSA treatment (≈160-fold enrichment). Moreover, Tau was barely detectable at the p21 transcription start site region in MCF7shctrl cells, expressing Tau proteins (less than 1.4-fold enrichment compared to IgG ctrl) (Figure 3D). qPCR analyses were then performed to detect the p21 proximal and distal regions. A significant increase in ac-H3 was observed in MCF7shTau cells (≈10-fold enrichment compared to non-treated cells) but not in MCF7shctrl cells (Figure 3C). In addition, Tau was detected at the level of these regions in MCF7shctrl cells (≈4-fold enrichment) in absence or presence of TSA treatment (Figure 3D). These results are consistent with our hypothesis that Tau4R modulated the TSA induced-histone acetylation by binding to chromatin.

We next performed chromatin immunoprecipitations in the GAL4UAS responsive luciferase reporter Hela stable cell line for which we observed similar gene response after exogenous Tau4R expression and 100 nM TSA treatment (see Figure 2F above). We performed qPCR as described previously to detect a fragment of the integrated promoter, encompassing the GAL4UAS responsive elements and the transcription start site (Figure 3E). A total of 100 nM TSA caused a 10-fold increase of ac-H3 in the control HeLa cells that was abrogated in the presence of transfected Tau4R. As expected, Tau4R was specifically enriched in the non-treated and TSA-treated conditions (7- and 16-fold, respectively).

Taken together, these data demonstrated that Tau bound various DNA regions and inhibited histone H3 TSA-induced acetylation.

### Tau4R Associates With Condensed Chromatin

**FIGURE 4:**
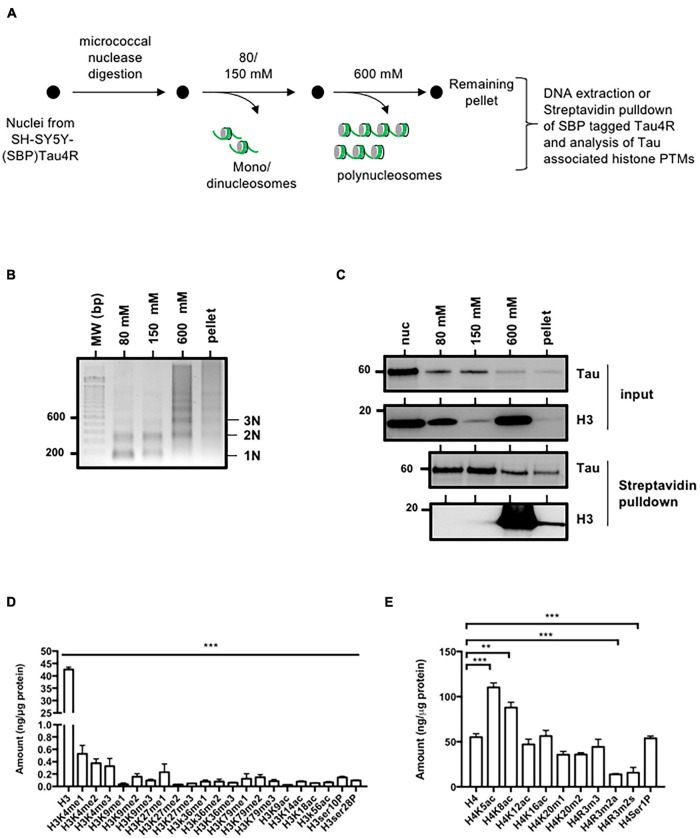
Tau4R is associated with condensed chromatin. **(A)** Schematic representation of salt fractionation of nucleosomes steps. Nuclei were isolated and digested with micrococcal nuclease and extracted successively with the indicated NaCl concentration. **(B)** DNA characterization from SH-SY5Y-(SBP)Tau4R cell chromatin fractions. DNA ladder obtained from micrococcal nuclease-treated nuclei purified as described in **(A)**. Nucleosomes are indicated on the right. **(C)** Tau interacts with histones in the condensed chromatin and the transcriptionally active fractions. Equal aliquots of each salt fraction were resolved on 12% SDS-PAGE and visualized by immunoblot analysis with antibodies against Tau or H3 (input). The remainder was used for streptavidin pulldown of SBP (streptavidin binding peptide) tagged Tau4R. Bound fractions were resolved on 12% SDS-PAGE and visualized by immunoblot analysis with antibody against H3. **(D)** Analysis of the different histone H3 or **(E)** H4 post-translational modifications associated with Tau in the condensed chromatin fraction (600 mM). Eluted Tau4R complex obtained from the 600 mM fraction obtained as described in **(A)** were analyzed for H3 and H4 post-translational modifications using ELISA kits. Data are mean ± SD, **P < 0.01, ***P < 0.001. All results are representative of three independent experiments.

Successive salt extraction of intact MNase-treated nuclei results in the isolation of chromatin fractions with different genome-wide profiles (Henikoff et al., 2009). The low salt soluble fraction contains active chromatin while the high-salt fraction is enriched in condensed/active chromatin. In addition, transcribed regions of the genome are found in the remaining pellet. We therefore sought insights into Tau4R’s distribution on chromatin. To do this we digested and fractionated stable SH-SY5Y cells overexpressing Tau4R fused in frame with the streptavidin binding peptide [SH-SY5Y-(SBP)Tau4R], allowing the elution of Tau4R-associated proteins (Figure 4A). As expected, DNA analyses showed that low salt fractions (80 and 150 mM) were mostly enriched in mononucleosomes, representing the active chromatin, while the high-salt fraction (600 mM) contained exclusively polynucleosomes, i.e., condensed chromatin (Figure 4B). In addition, western-blot analysis demonstrated that Tau was detected in all the fractions tested, as was histone H3 (Figure 4C, input). We next wondered if Tau4R might be specifically interacting with nucleosomes in the different chromatin states. For this, we performed streptavidin pulldown of the Tau fusion and tested the presence for Tau itself and that of nucleosomes (by the presence of H3) by western-blot analysis. As shown in Figure 4C, immunoprecipitated H3 was almost exclusively in the high-salt fraction while Tau was more evenly present in all fractions. These results suggested that Tau was preferentially associated with condensed chromatin. Several hypotheses could explain this. Previous reports demonstrated that Tau can bind DNA *in vitro* and could be associated with GAGA responsive elements (GAGA-RE) (Benhelli-Mokrani et al., 2018). However, we found no preferential binding for GAGA-RE compared to control sequence using microscale thermophoresis (Supplementary Table 1). We therefore next considered the possibility that Tau could be associated with condensed chromatin because of post-translational modifications of histones, highly specific to the different chromatin compartments. After streptavidin pulldown of SBP-Tau4R, bound materials were eluted with biotin and tested for the presence of post-translational modifications of H3 and H4 with commercial ELISA kits. Non-modified H3 was present but none of the tested post-translational modifications were detected (Figure 4D). For H4 there was almost no signal for H4R3me3a and m2s. However, all the other tested modifications were strikingly present in the Tau immunoprecipitate (Figure 4E). Note that unmodified H4 signal was weaker than certain H4 post-translational modifications, suggesting that they might interfere with total H4-ab binding. However, normalization against total H3 did not modify the obtained results.

Taken together, these data place Tau4R in condensed chromatin regions containing acetylated H4 and unmodified H3.

### Tau4R/Chromatin Interaction Is Mediated Through Histones

**FIGURE 5:**
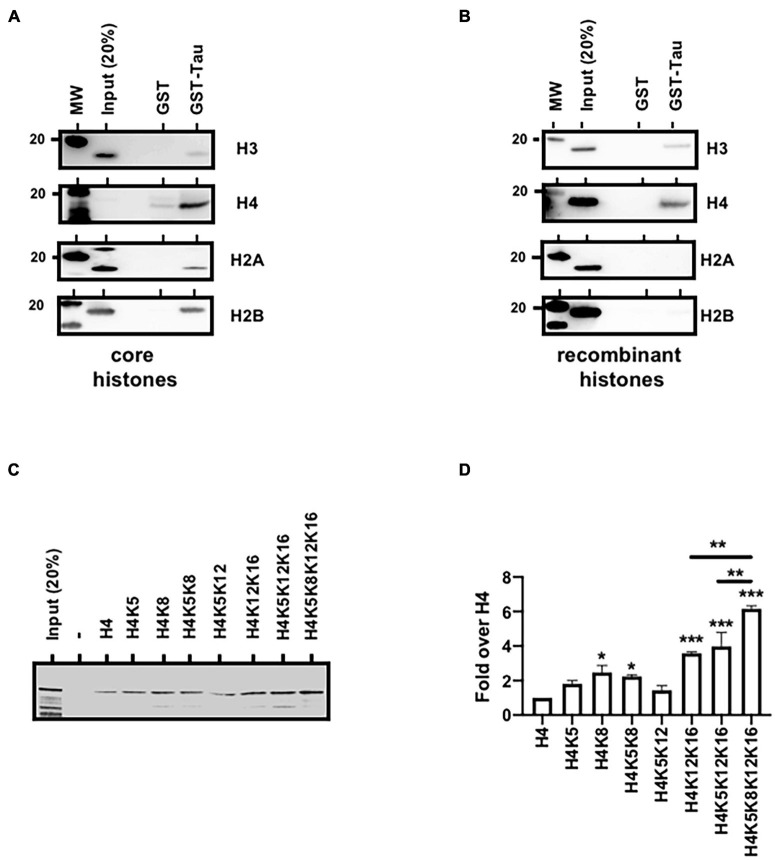
Tau4R binds directly to histones. **(A)** Tau4R interacts with core histones. GST-pulldown experiments were carried out using GST (1 μg) or GST-Tau4R (1 μg) and purified core histones (1 μg). Complexes were precipitated with Sepharose-glutathione beads, resolved by 12% SDS-PAGE and visualized by immunoblot analysis with antibodies against H3, H4, H2A, and H2B. **(B)** Tau4R interacts with histone H3 and H4. GST-pulldown experiments were carried out using 1 μg of GST or GST-Tau4R and 1 μg of recombinant H3, H4, H2A, or H2B. Complexes were precipitated with Sepharose-glutathione beads, resolved by 12% SDS-PAGE and visualized by immunoblot analysis with antibodies against H3, H4 H2A, or H2B. **(C)** Histone H4 tail peptides tested for Tau binding. Tau protein bound to biotinylated synthetic H4 peptides was detected by immunoblot. A representative western blot is shown and **(D)** is a quantification from three independent experiments. Data are mean ± SD, *P < 0.05, **P < 0.01, ***P < 0.001.

Naturally our next hypothesis then was that tau could be associated with chromatin through direct interaction with histones. To address this possibility, we first realized GST-pulldown analysis using unmodified core histones. As shown in Figure 5A, Tau4R but not GST alone bound specifically to the histone core as revealed by H3, H4, H2A, and H2B antibodies. Using recombinant histones, we also showed that this interaction was mediated through H3 and/or H4 and not H2A and H2B (Figure 5B). We next tested the effect of different combination of H4 acetylation sites, not present in the H4 ELISA kit, on Tau4R interaction using synthetic H4 tail peptides. As seen in Figures 5C,D, Tau bound to unacetylated H4 and H4K5acK12ac. In contrast to H4K5ac, acetylation at K8 (H4K8) slightly increased Tau binding (twofold over unacetylated H4). Tau binding was not further increased by acetylation at position 5 (H4K5acK8ac). We observed a fourfold increase in Tau binding for diacetylated H4 at position 12 and 16. Again, no change was observed when adding an acetyl group at position 5. However, a sixfold increase was observed when using a tetra-acetylated H4. These results indicated that Tau binds H4 when unacetylated, but displays more affinity for tetra-acetylated H4.

### The Frontotemporal Lobar Degeneration Tau Mutation Abolished Tau/Histone Interaction

**FIGURE 6:**
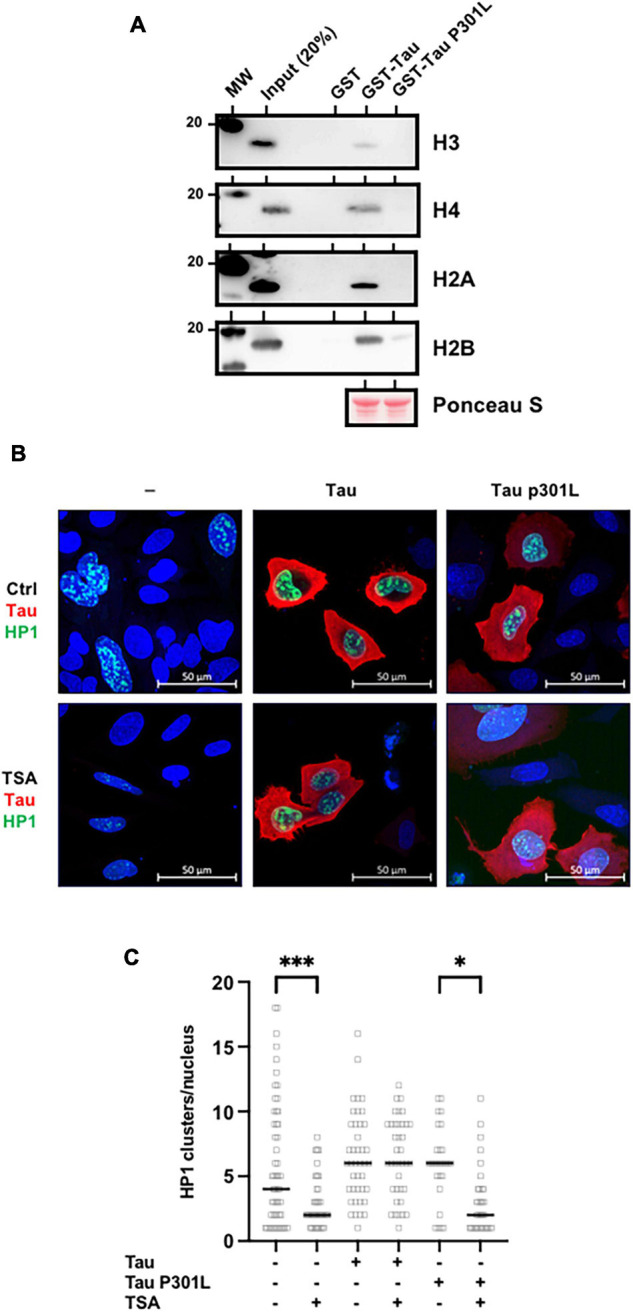
Frontotemporal lobar degeneration Tau mutations disrupt its interaction with histones. **(A)** P301L mutation abolished Tau/histone interaction. GST-pulldown experiments were carried out using GST (1 μg), GST-Tau4R (1 μg) or GST-P301L (1 μg), and purified core histones (1 μg). Complexes were precipitated with Sepharose-glutathione beads, resolved by 12% SDS-PAGE and visualized by immunoblot analysis with antibodies against H3, H4, H2A, and H2B. GST-Tau and GST-TauP301L loading was controlled by Ponceau red staining. **(B)** Single confocal sections of Hela cells transfected with GFP-HP1β, with or without Tau4R or TauP301L, and treated 24 h later with the TSA (300 nM) for 24 h. Tau C-terminus antibodies and GFP fluorescence were used to visualize total Tau protein and HP1β respectively. Representative images are shown. **(C)** Quantification of HP1β clusters per nuclei visualized as described previously and realized on three independent experiments. Data are mean ± SD, ^∗^*P* < 0.05, ^∗∗∗^*P* < 0.001.

Pericentromeric heterochromatin disruption was observed in neurons from frontotemporal lobar degeneration (Tau P301L/S) pathological models (Frost et al., 2014; Mansuroglu et al., 2016). These observations further suggest that P301L/S mutations, could nevertheless abolish Tau/histone interaction. To this end, we first performed GST-pulldown analysis using purified core histones. As shown previously, Tau interacted specifically with core histones as detected by H3, H4, H2A, and H2B antibodies (Figure 6A). However, this interaction was greatly decreased by the TauP301L mutation. Based on these observations, we hypothesized that TauP301L mutant would not prevent HP1 spreading induced by TSA treatment. To test this, we next followed the fate of transfected GFP-HP1β, in the absence or presence of transfected Tau4R or TauP301L mutant, following TSA treatment in Hela cells, which as we have mentioned are devoid of endogenous Tau expression. As shown above, we observed a decrease in the number of HP1 clusters in control cells following TSA treatment. This decrease was prevented when wild-type Tau was expressed, but not by the Tau P301L mutant (Figures 6B,C).

Taken together, these data demonstrated a direct relationship between Tau/histone interaction and TSA-induced chromatin remodeling.

### Tau4R Increases Chromatin Compaction

A possible mechanism of the inhibitory effect on H3 acetylation is that Tau4R binding to histone blocks acetylation by steric hindrance. To test this hypothesis, we next performed *in vitro* acetyltransferase assays using core histone, unmodified or H4 tetra-acetylated nucleosomes. However, no change in H3 or H4 acetylation level was observed in the absence or presence of recombinant Tau4R (Supplementary Figure 6).

Another intriguing possible mechanism is that Tau4R binding to histones would induce chromatin compaction that prevents H3 acetylation. To address this question, we again used our micrococcal nuclease assay as the outcome is highly dependent on chromatin compaction. It has been shown that the cleavage pattern as well as the rate of conversion of chromatin into smaller nucleosome fragments reflected the accessibility of linker DNA to the enzyme and overall compactness of chromatin (Bryant, 2012).

**FIGURE 7:**
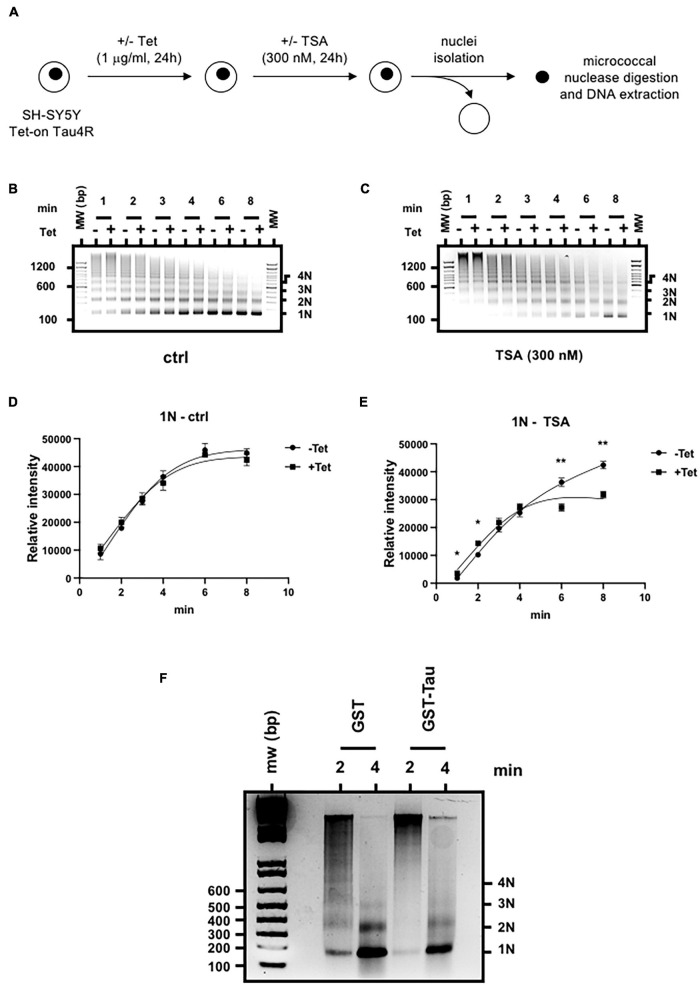
Tau4R increases chromatin compaction. **(A)** Schematic representation of the experimental procedure. Nucleosome ladders obtained after micrococcal nuclease digestion of SH-SY5Y Tet-on Tau4R cells treated or not with tetracycline (Tet, 1 μg/ml, 24 h) then followed **(B)** or not **(C)** by TSA treatment (300 nM, 24 h). Digested chromatin was analyzed on a 1.5% agarose gel and revealed by ethidium bromide staining. Mono-, di-, tri, and tetra-nucleosomes are indicated on the right. One representative experiment out of three is shown. **(D,E)** Densitometric analysis of the 1N fragments obtained in control **(B)** and TSA-treated conditions **(C)** were calculated from three independent experiments. Data are mean ± SD,*P < 0.05, **P < 0.01. **(F)** Tau4R reduces chromatin template accessibility to micrococcal nuclease *in vitro*. Reconstituted nucleosomal arrays were incubated with 3 μg of GST or GST-Tau4R and subjected to micrococcal nuclease digestion for 2 or 4 min. Purified DNA fragments were then run on a 1.5% agarose gel in 0.5× TBE buffer and revealed by ethidium bromide staining. Mono-, di-, tri, and tetra-nucleosomes are indicated on the right. One representative experiment out of three is shown.

To probe the effect of Tau on chromatin structure, this time we performed the nuclease assay using different time points. To control Tau4R expression, we therefore used SH-SY5Y Tet-on Tau4R cells. We induced Tau4R expression with tetracycline and then added 300 nM TSA, 24 h later (Figure 7A). Then, we subjected an equivalent number of isolated nuclei to micrococcal nuclease digestion for different times (1, 2, 3, 4, 6, and 8 min) and compared the abundance of nucleosomal DNA corresponding to mono-, di-, tri-, and tetranucleosomes. In the absence of TSA, chromatin from control and Tau4R expressing cells cleaved in a similar pattern at a similar rate (Figures 7B,D and Supplementary Figure 7). Importantly, however, TSA induced significant differences. There were less oligo-nucleosomes in Tau4R expressing cells, demonstrating that the chromatin was less accessible to micrococcal nuclease, meaning more compact, when Tau4R was present (Figures 7C,E and Supplementary Figure 7).

To confirm our results were not due to experimental artifacts in our cell lines, we next used *in vitro* reconstituted chromatin incubated with purified recombinant GST or GST-Tau4R proteins in a micrococcal nuclease assay. As shown in Figure 7F, digestion with GST alone for 2 min produced a ladder of DNA fragments, mostly corresponding to mono-, di-, tri-, and tetra-nucleosomes whereas the longer incubation (4 min) gave rise mostly to mono and di-nucleosomes. Strikingly, when *in vitro* reconstituted chromatin was pre-incubated with purified GST-Tau4R, no ladder was observed after 2- or 4-min incubation with micrococcal nuclease, showing that chromatin in this condition was less accessible. These observations are consistent with a role of Tau4R in maintaining a compacted chromatin structure that prevents histone acetylation.

## Discussion

An extensive body of literature suggested a possible role of Tau in chromatin functions and/or organization in neuronal, non-neuronal cells, and cancer cells (Frost et al., 2014; Bukar Maina, 2016; Bou Samra et al., 2017; Klein et al., 2019). However, a clear mechanism has not been demonstrated. Here we show that Tau preferentially binds to the condensed nuclease-resistant chromatin fraction devoid of any silent specific histone methylation. In addition, we demonstrated that Tau binding to histones is direct, involving unmodified histone H3 and H4 or tetra-acetylated H4 that increased interaction. As consequences, Tau 4R stabilizes condensed chromatin and heterochromatin.

Our results point out that Tau expression itself does not induce broad changes in chromatin organization/structure (e.g., chromatin accessibility, pericentromeric heterochromatin integrity, and histone acetylation) and the effect of Tau was seen only when deacetylation was inhibited. These data suggest a more general role for Tau in preventing chromatin remodeling. In this regard, we found the same results using BIX 01294, a specific inhibitor of G9a histone methyltransferase catalyzing the di-methylated state of H3 at lysine 9 and known to disrupt pericentromeric heterochromatin (Kubicek et al., 2007; Supplementary Figure 8). Most strikingly, associated histones were devoid of any H3K9me2/3, a hallmark of heterochromatin. We found that Tau4R was able to maintain PCH structure irrespectively of the observed decrease in H3K9me2 induced by TSA or BIX 01294, suggesting an indirect role for Tau4R in maintaining PCH and heterochromatin integrity. The mechanism by which TSA reduced the level of this epigenetic mark remains unknown but has been reported previously (Fukuda et al., 2015). In a drosophila model of tauopathy, heterochromatin disruption was hypothesized to be a consequence of oxidative stress/DNA damage and this effect could not be prevented by aggregated Tau proteins. Interestingly, there is now evidence suggesting that oxidative stress globally influences chromatin structure and enzymatic post-translational modifications of histones (Kreuz and Fischle, 2016). These observations suggest that loss of Tau function and chromatin remodeling act at the initial step of the pathology.

In drosophila, global changes in gene expression have been also observed, indicating chromatin remodeling is not limited to pericentromeric heterochromatin structure. These observations were recapitulated in part in our cellular models. Trichostatin-induced gene expression was higher in cellular MCF7 models depleted of endogenous Tau for GADD45a and p21, two well-characterized histone deacetylase-inhibitor-inducible genes. In addition, luciferase reporter activation was observed after trichostatin A treatment and decreased in the presence of GAL4-tethered or wild-type Tau4R proteins. Note also that we did not observe an effect of GAL-Tau4R when the GAL4UAS responsive luciferase reporter Hela stable cell line displayed a high basal luciferase, representing state associated with active chromatin (Supplementary Figure 9). This observation suggests that Tau4R was unable to repress gene expression on active genes but rather maintains the condensed chromatin state.

Our results clearly demonstrated that Tau was mostly associated with condensed chromatin, where associated histones were devoid of any H3 post-translational modifications and could present in particular H4 acetylation marks. Our *in vitro* experiments demonstrate that Tau4R binds to unmodified core histones, but the affinity increased with H4 acetylation. Using genome-wide chromatin immunoprecipitation followed by microarray hybridization assays in primary neuronal culture, Benhelli-Mokrani et al. (2018) suggested that an AG-rich GAGA-like DNA motif could play a role in Tau genomic localization. However, we found no correlation between the presence of GAGA sequences and Tau binding (Supplementary Table 1). Our observation is more consistent with a previous report demonstrating that Tau DNA binding is sequence independent, involving just the DNA backbone (Qi et al., 2015). Sequence-independent DNA binding cannot explain the observed specific genomic distribution observed by Benhelli-Mokrani et al. (2018). The interaction with histones and nucleosome core particles we reveal likely confer additional specificity for Tau binding to chromatin.

The exact modality of Tau4R binding to histones H3 and H4 remains to be identified, since no structural similarities with other histone associated proteins were found. Since we did not observe any chromatin association with the Tau3R isoforms, we suspect an essential role of the second microtubule domain encoded by the exon 10 in the recognition process. This observation is reinforced by the loss of Tau/histone interaction observed with the P301L mutant. The mutation occurs within exon 10 and only affects Tau4R isoform, exon 10 being spliced out of 3R isoforms (Sergeant et al., 2005). Note that the TauP301L/S mutations do not interfere with Tau nuclear localization (Siano et al., 2019).

Although Tau contains an intrinsic acetyltransferase activity, it did not appear to contribute directly to this specific acetylation pattern as demonstrated by our *in vitro* acetyltransferase assays (Cohen et al., 2013). It was quite surprising to find H4K16 acetylation in these nuclease resistant fractions, as this is a histone post-translational modification known to contribute directly to chromatin decompaction (Shogren-Knaak et al., 2006; Yu et al., 2011). However, there is substantial evidence that H4K16 acetylation does not alter higher chromatin compaction *in vivo* but rather it disrupts local chromatin structure (Taylor et al., 2013; Mishra et al., 2016). In addition, H4 acetylation has been detected in some heterochromatin compartments (Turner et al., 1992; Johnson et al., 1998). Most strikingly, our results show that Tau associated histones were devoid of the H3K9me2/3 hallmark of heterochromatin. This further suggests an indirect role for Tau4R in maintaining heterochromatin integrity.

**FIGURE 8:**
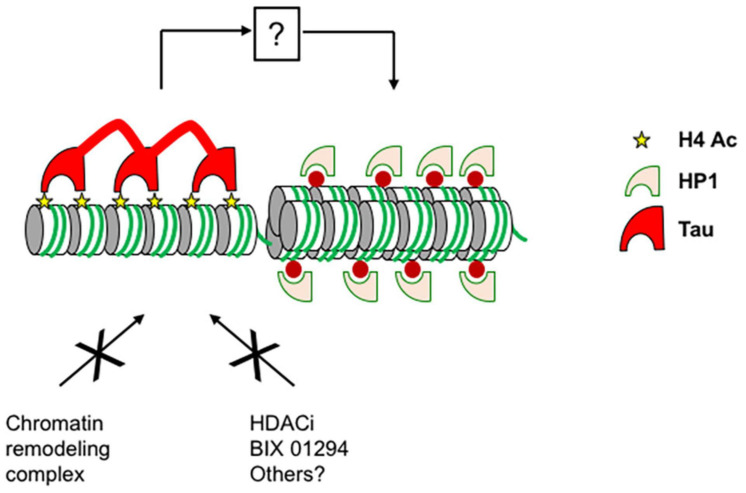
Proposed model for the role of Tau on chromatin. Tau binds to condensed chromatin regions through the interaction with unmodified histone H3, H4, or acetylated H4. The interaction between Tau and histones maintains the condensed chromatin state and prevents chromatin remodeling complexes accessing histones and gene expression. By an indirect mechanism, Tau also prevents heterochromatin decompaction and HP1s spreading induced by chromatin remodeling agents such as TSA, BIX 01294, and possibly others.

Our results presented here indicate that Tau binding specifically inhibits H3 acetylation *in cellulo*, as shown for the p21 promoter (distal and proximal) in MCF7 cells and for the GAL4UAS responsive luciferase reporter in Hela cells. Tau was not detected at the level of p21 transcription start site, a region shown to be enriched in H3K4me3, which supports our idea that post-translational modification of H3 prevents Tau binding (Itahana et al., 2016). However, this effect was not seen not for nucleosomes or histones *in vitro*. On the balance of the data presented here we feel that the chromatin’s condensation state prevents chromatin remodeling complexes accessing histones (Figure 8). Although our experiments demonstrate that histone acetylation is inhibited by Tau, other post-translational modifications of H3 are also likely inhibited as there are not seen in our streptavidin pull-down assay.

As a consequence of a cellular role of Tau in chromatin organization and/or function, we showed that Tau4R depletion in the luminal MCF7 or triple-negative MDA-MB-231 breast cancer cell lines increased TSA-induced cell death and apoptosis. Previous studies demonstrated that histone deacetylase-inhibitors led in particular to the up-regulation of pro-apoptotic genes (Li and Seto, 2016). We found that GADD45a, a gene that may be involved in apoptosis, was more expressed in MCF7shTau cells after TSA treatment (Takekawa and Saito, 1998; Zhang et al., 2001). Although p21 is known to be induced by inhibiting histone deacetylases, it is not clearly known how it controls the resulting apoptosis, although p21 can induce G1 arrest. Depending on the cellular model, G1 arrest could be protective or necessary for TSA-induced apoptosis (Peart et al., 2005; Newbold et al., 2014). In this regard, we found no correlation between p21 expression, G1 arrest and the extent of apoptosis in MCF7 and MDA-MB-231 short hairpin cells.

In conclusion, this is the first study describing a role and underlying mechanism of Tau protein in chromatin structure and opens new avenues to further understand Tau biology in neuronal and cancer cells.

## Data Availability Statement

The original contributions presented in the study are included in the article/Supplementary Material, further inquiries can be directed to the corresponding author.

## Author Contributions

BL designed the research. TR, MG, AC, TC, MC, HD, FP, and BL performed the experiments. RM and XT performed the microscale thermophoresis experiments and analyzed the data. TR, MG, AC, FP, LB, and BL analyzed the data. BL wrote the manuscript with the input of LB and M-CG. All authors contributed to the article and approved the submitted version.

## Conflict of Interest

The authors declare that the research was conducted in the absence of any commercial or financial relationships that could be construed as a potential conflict of interest.

## Publisher’s Note

All claims expressed in this article are solely those of the authors and do not necessarily represent those of their affiliated organizations, or those of the publisher, the editors and the reviewers. Any product that may be evaluated in this article, or claim that may be made by its manufacturer, is not guaranteed or endorsed by the publisher.
