# Supplementary material for: Tau Stabilizes Chromatin Compaction
Source: Front Cell Dev Biol. 2021 Oct 14;9:740550. doi: 10.3389/fcell.2021.740550 (PMC8551707; doi:10.3389/fcell.2021.740550)
Supplement: Supplementary file 1 [file Data_Sheet_1.PDF]

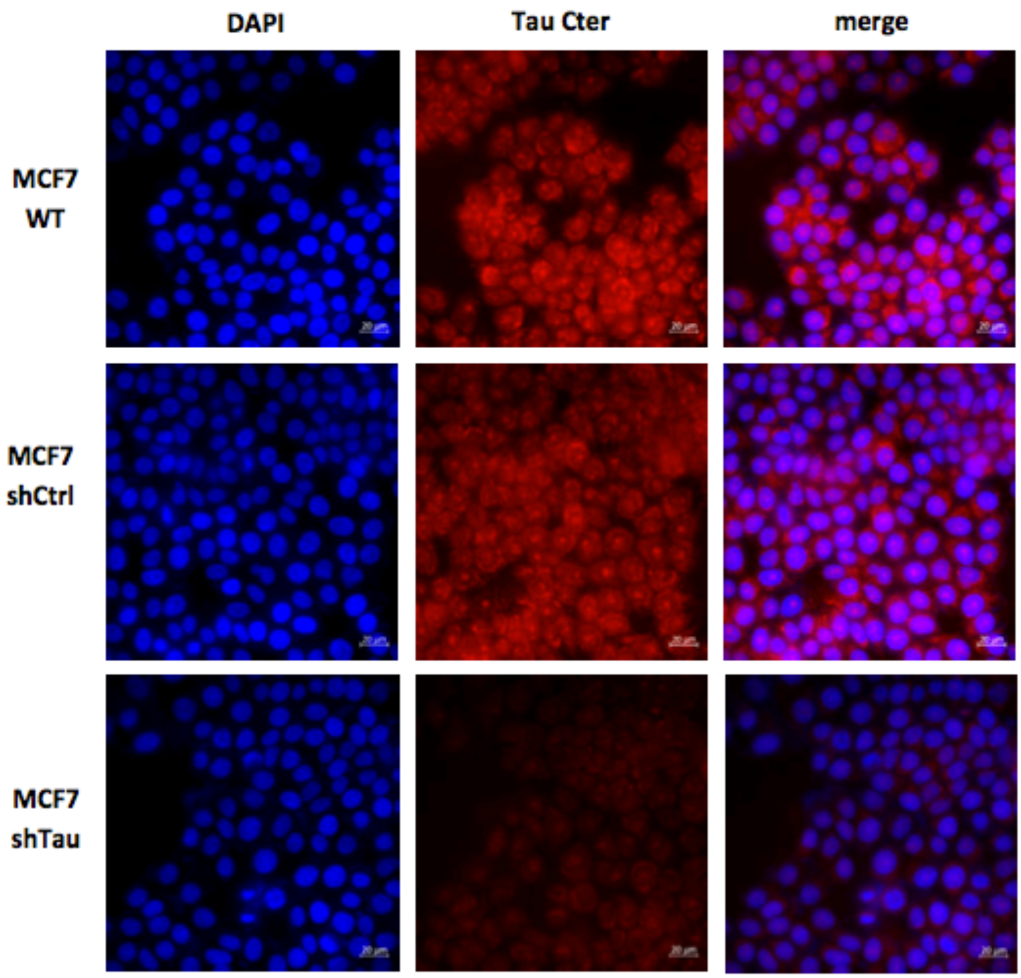

Supplementary Figure 1 : Single confocal sections of MCF7 wt, MCF7 stably transfected with plasmids encoding shctrl or shTau. Tau C-ter antibody was used to visualize total Tau protein. Representative images are shown.
