# Supplementary material for: Tau Stabilizes Chromatin Compaction
Source: Front Cell Dev Biol. 2021 Oct 14;9:740550. doi: 10.3389/fcell.2021.740550 (PMC8551707; doi:10.3389/fcell.2021.740550)
Supplement: Supplementary file 2 [file Data_Sheet_2.PDF]

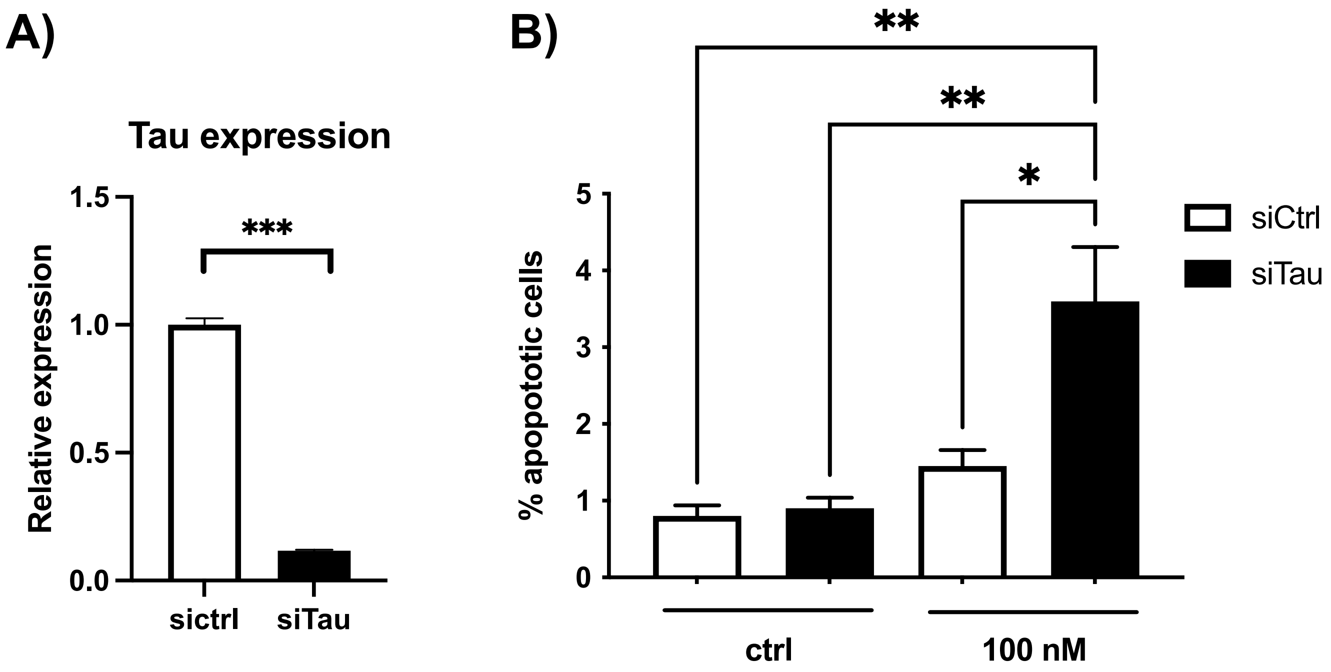

Supplementary Figure 2 : Tau inhibition by siRNA increases TSA-induced apoptosis in MCF7 breast cancer cell line.

(A) Tau expression in MCF7 cells transfected with a siRNA scramble (sictrl) or a siTau. Tau expression was analyzed by real-time PCR and normalized to RPLO three days after transfection. Results are expressed, relative to the basal activity set to 1, as the mean ± SD of three independent assays. (B) Effect 100 nM TSA (24h) on apoptosis in the MCF7 cells transfected with a siRNA scramble (sictrl) or a siTau. MCF7 cells were transfected with a siRNA scramble (sictrl) or a siTau then three days later treated or not with 100 nM TSA for 24h. Apoptosis was determined by flow cytometric analysis of the PI-positive and Annexin-V-positive cells as described in the materials and methods.
