# Supplementary material for: Tau Stabilizes Chromatin Compaction
Source: Front Cell Dev Biol. 2021 Oct 14;9:740550. doi: 10.3389/fcell.2021.740550 (PMC8551707; doi:10.3389/fcell.2021.740550)
Supplement: Supplementary file 3 [file Data_Sheet_3.PDF]

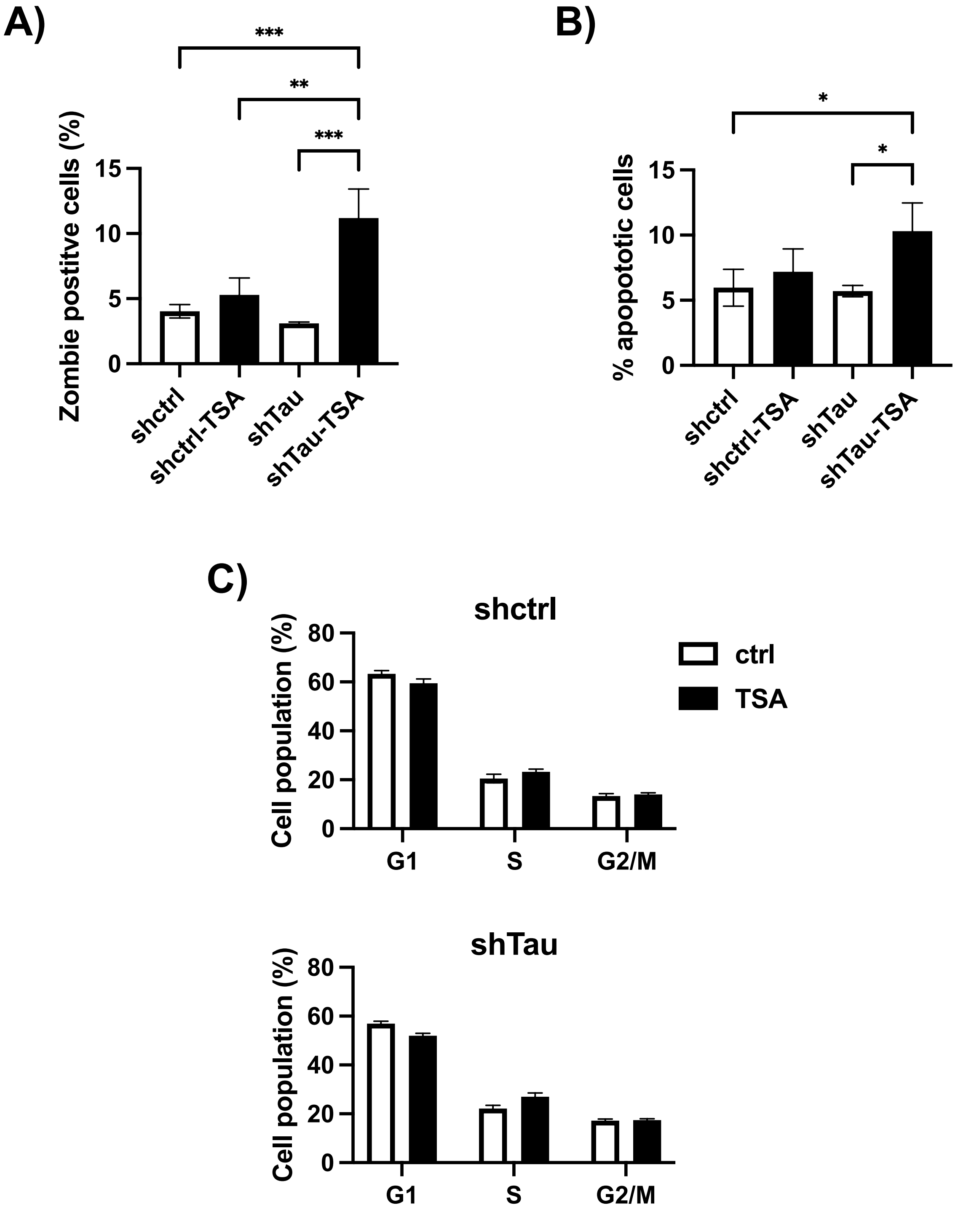

Supplementary Figure 3 : Tau inhibition increases MDA-MB-231 breast cancer cell line sensitivity to TSA. (A) Effect of 100 nM TSA treatment (48h) on cell death in MDA-MB-231shctrl and MDA-MB-231shTau subclones. Cell death was determined by zombie staining and flow cytometric analysis as described in the materials and methods. (B) Effect of 100 nM TSA treatment (48h) on apoptosis in in MDA-MB-231shctrl and MDA-MB-231shTau subclones. Apoptosis was determined by flow cytometric analysis of the PI-positive and Annexin-V-positive cells as described in the materials and methods. (D) Cell cycle distribution was determined by FACS analysis of combined propidium iodide and EdU staining in MDA-MB-231shctrl and MDA-MB-231shTau subclones in the absence or presence of 100 nM TSA, 48h. Data are mean±SD. *P<0.05***P<0.001. All results are representative of three independent experiments.
