# Supplementary material for: Tau Stabilizes Chromatin Compaction
Source: Front Cell Dev Biol. 2021 Oct 14;9:740550. doi: 10.3389/fcell.2021.740550 (PMC8551707; doi:10.3389/fcell.2021.740550)
Supplement: Supplementary file 4 [file Data_Sheet_4.PDF]

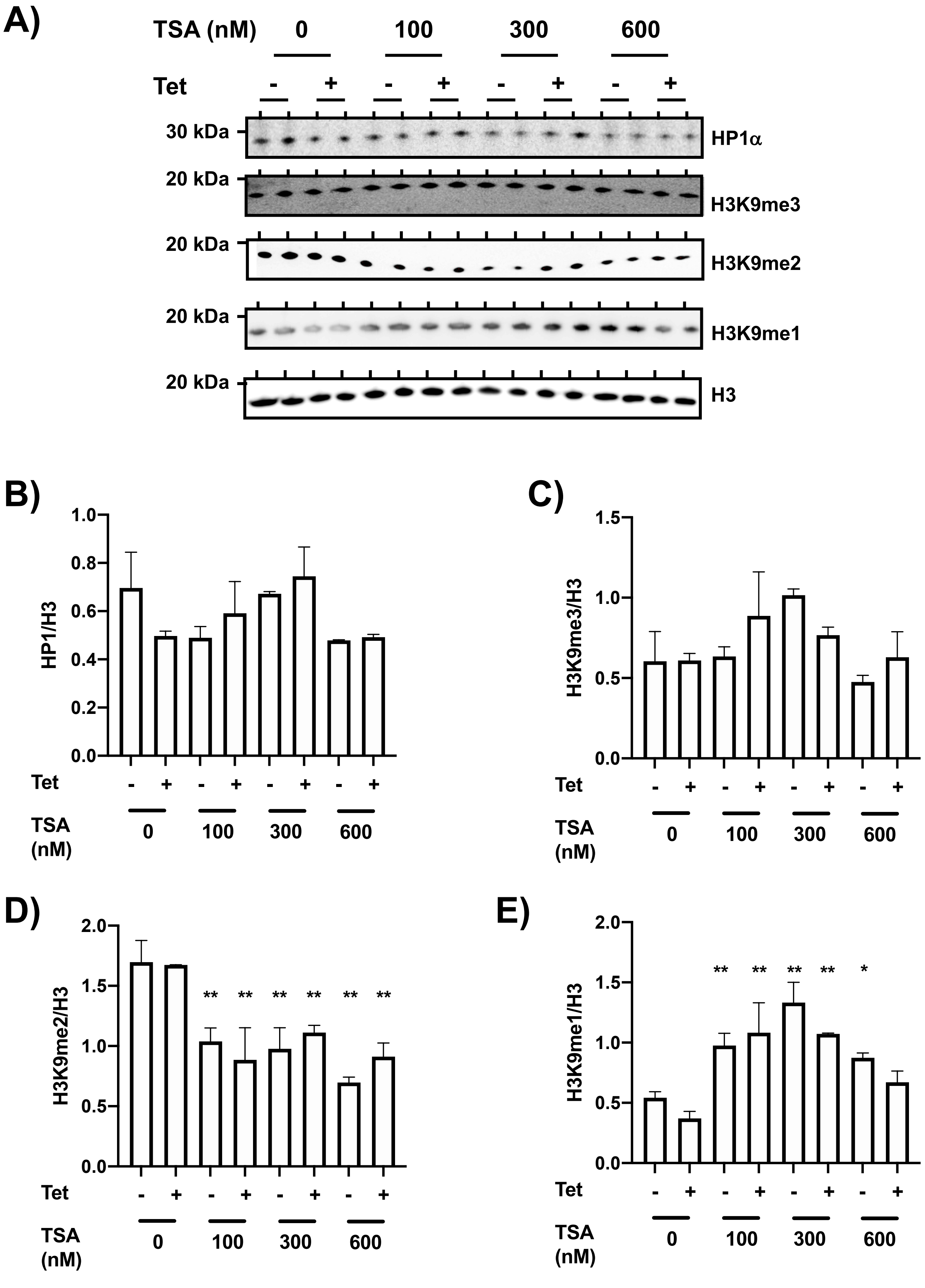


**Supplementary Figure 4 : Rate of H3K9 methylation and HP1α expression after TSA treatment.** (A) Western-blot analysis of HP1α, H3K9me3, H3K9me2 and H3K9me1 in SH-SY5Y Tet-on Tau4R cells following TSA treatment. Proteins from whole cell extracts were resolved on 12% SDS-PAGE and visualized by immunoblot analysis with antibodies against HP1α, H3K9me2, H3K9me 3 and H3 antibodies. Quantification of HP1α (B), H3K9me3 (C), H3K9me2 (D) and H3K9me1 (E) in SH-SY5Y Tet-on Tau4R cells following TSA treatment revealed no difference between control and TSA treated cells. Data are mean±S.E.M. **P*<0.05, ***P*<0.01 vs control.
