# Supplementary material for: Tau Stabilizes Chromatin Compaction
Source: Front Cell Dev Biol. 2021 Oct 14;9:740550. doi: 10.3389/fcell.2021.740550 (PMC8551707; doi:10.3389/fcell.2021.740550)
Supplement: Supplementary file 5 [file Data_Sheet_5.PDF]

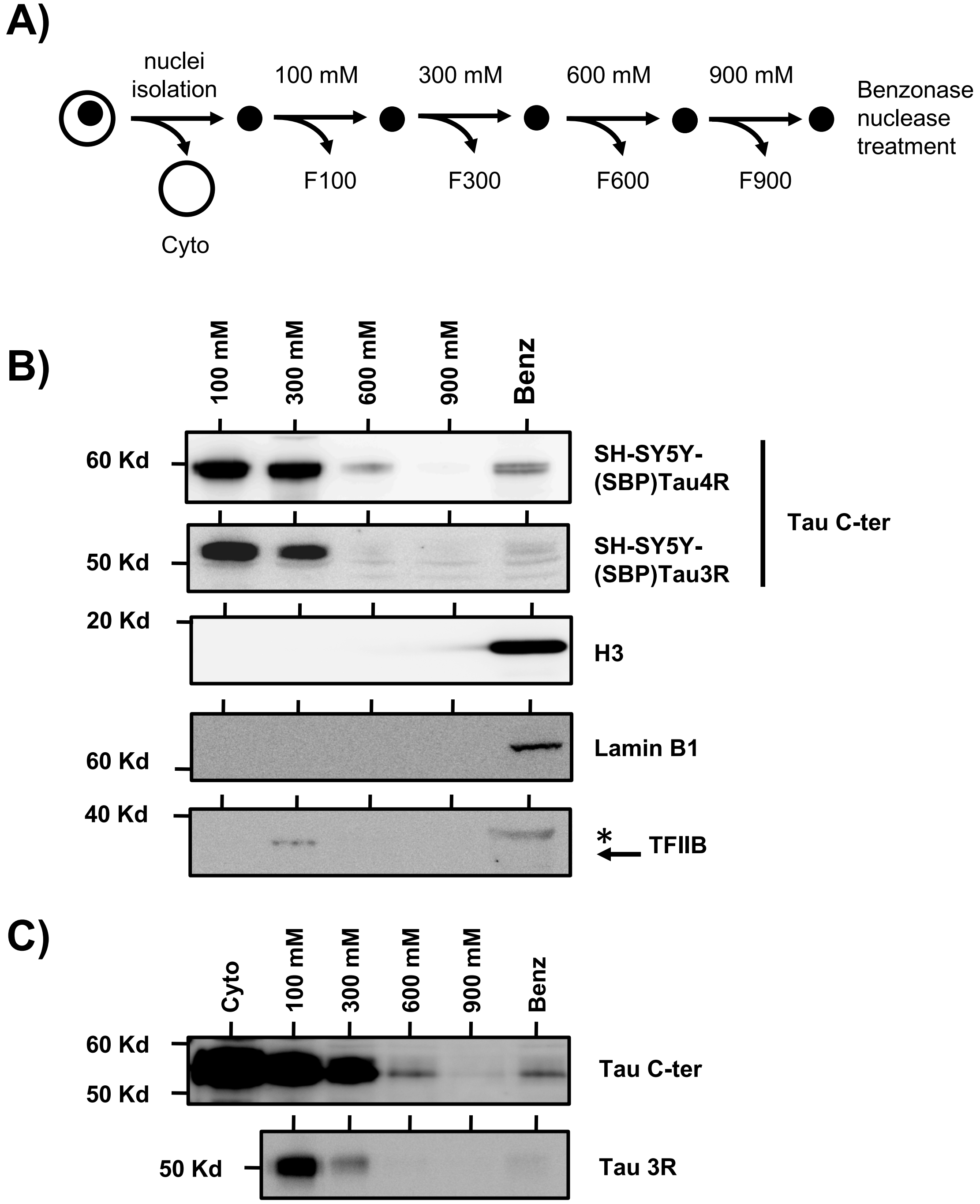

Supplementary Figure 5 : Tau4R but not Tau3R isoforms are tightly bound to chromatin.

(A) Schematic representation of cells fractionation steps. Nuclei were isolated and extracted successively with the indicated NaCl concentration. The final pellet was digested with Benzonase nuclease. (B) Solubility of Tau4R and 3R in differential salt extraction. Nuclei of stably transfected SH-SY5Y with Tau4R (SH-SY5Y-(SBP)Tau4R) or Tau3R (SH-SY5Y-(SBP)Tau3R) isoforms fused in frame with the streptavidin binding peptide (SBP) were extracted as indicated in A) and Tau was detected by immunoblot using Tau C-ter antibody. Representative blots obtained for SH-SY5Y-(SBP)Tau4R or SH-SY5Y-(SBP)Tau3R cells of protein controls known to be associated with the different fractions (Lamin B1 for the chromatin fraction/nuclear matrix fraction; TFIIB for the nuclear fraction, * non specific band). (B) Endogenous Tau isoforms were extracted from primary neuronal cells as described in (A) and detected using Tau C-ter antibody or specific to 3R isoforms.. All results are representative of three independent experiments.
