# Supplementary material for: Tau Stabilizes Chromatin Compaction
Source: Front Cell Dev Biol. 2021 Oct 14;9:740550. doi: 10.3389/fcell.2021.740550 (PMC8551707; doi:10.3389/fcell.2021.740550)
Supplement: Supplementary file 6 [file Data_Sheet_6.PDF]

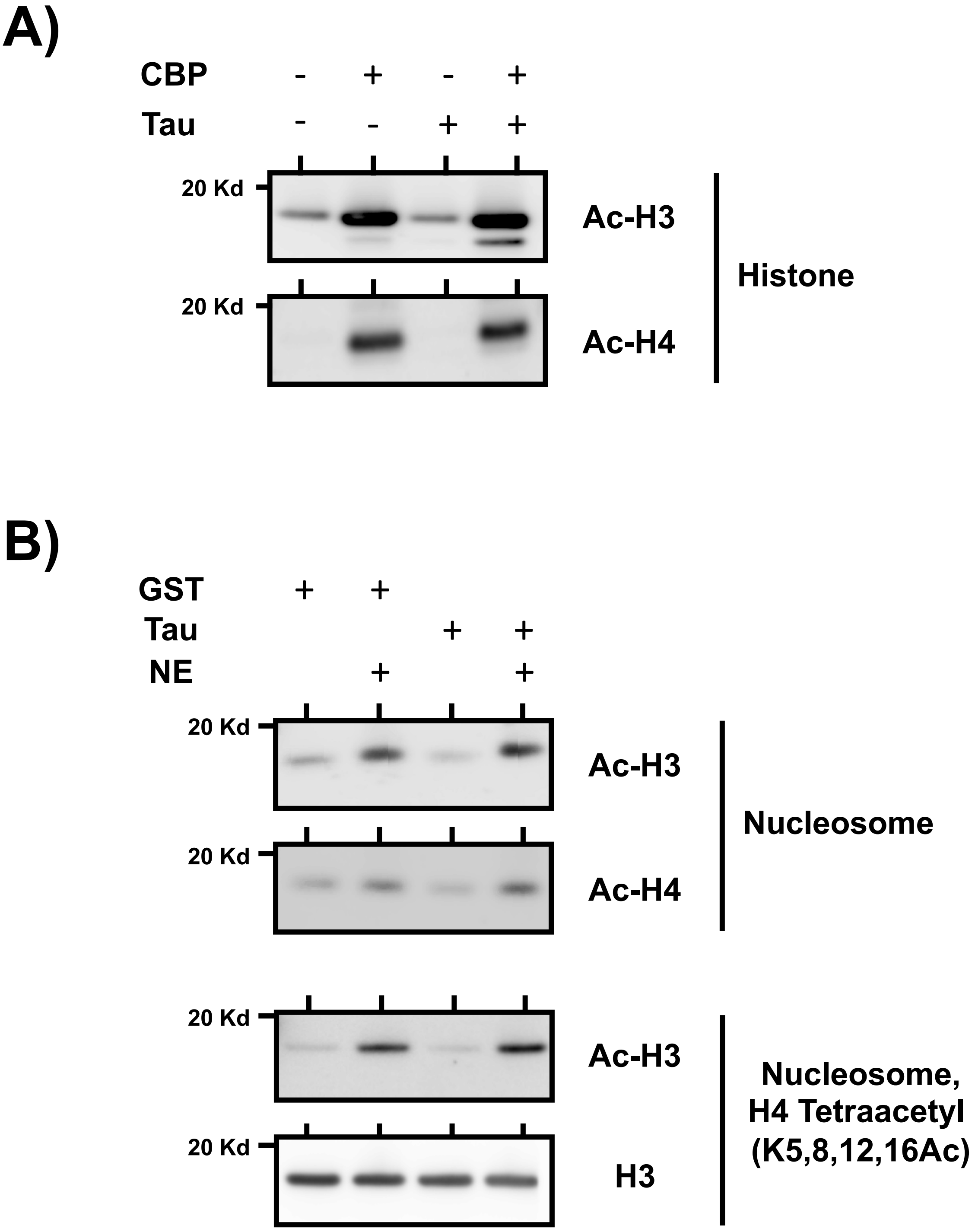

Supplementary Figure 6 : Tau did not prevent H3 acetylation *in vitro*.

Purified GST-CBP (A) or nuclear extracts from Hela cells (B) were incubated with histones, nucleosomes or tetraacetylated H4 nucleosome in the absence or presence of purified GST-Tau proteins. HAT assay was essentially performed as described previously (Tropberger, 2013).
