# Supplementary material for: Tau Stabilizes Chromatin Compaction
Source: Front Cell Dev Biol. 2021 Oct 14;9:740550. doi: 10.3389/fcell.2021.740550 (PMC8551707; doi:10.3389/fcell.2021.740550)
Supplement: Supplementary file 7 [file Data_Sheet_7.PDF]

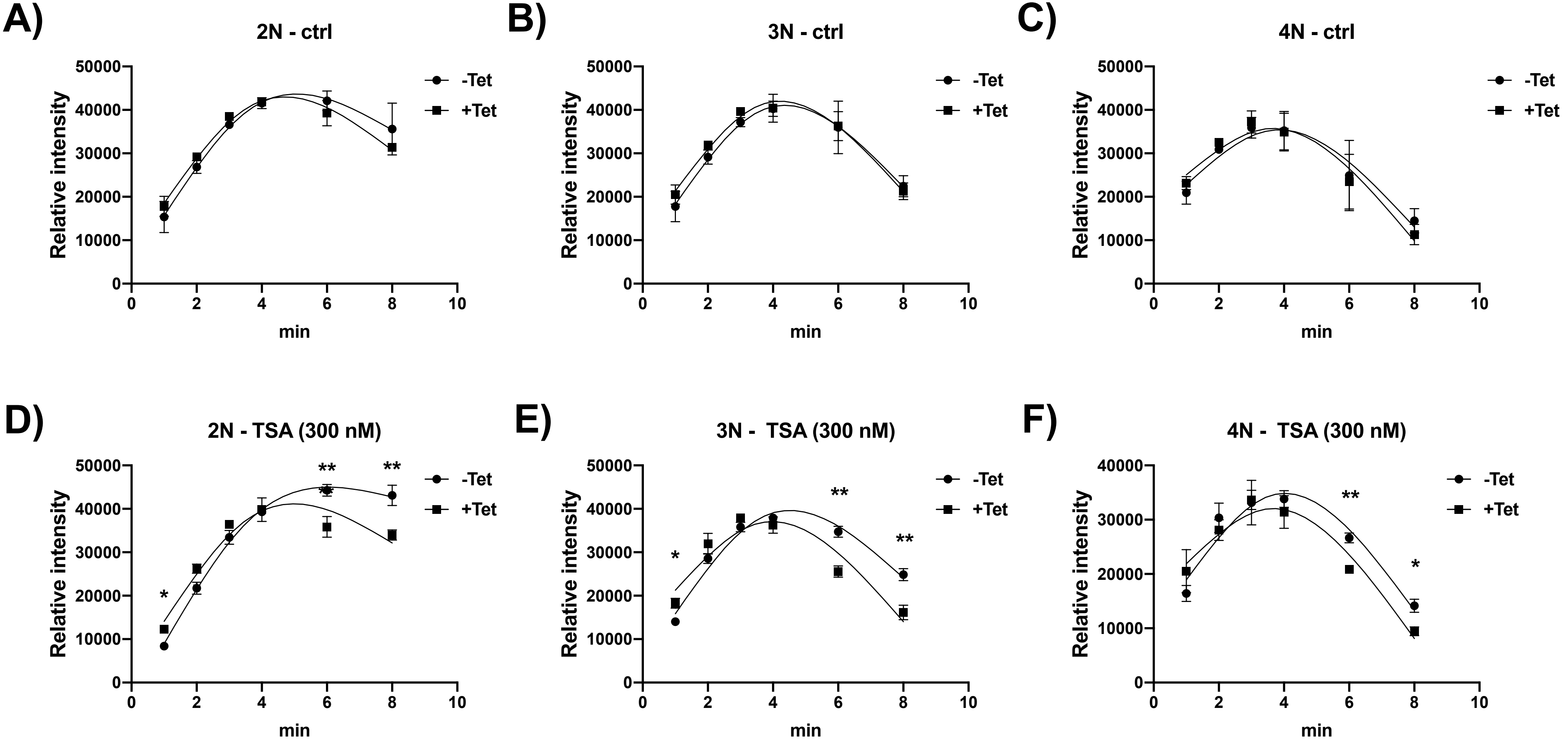

Supplementary Figure 7 : Chromatin of Tau expressing cells is less accessible to MNase after TSA treatment.

Chromatin from control SH-SY5Y Tet-on cells (A, B, C) or TSA treated (300 nM, 24h) (D, E, F) with Tau expression induced or not by tetracycline (Tet), 24h before TSA treatment, were analyzed at different time points. Densitometric analysis, calculated on three independent experiments, revealed substantially decreased intensity of the 2N, 3N and 4N fragments in Tau expressing cells after TSA treatment indicating a less accessible chromatin. Data are mean±SD *P<0.05, **P<0.01.
