# Supplementary material for: Tau Stabilizes Chromatin Compaction
Source: Front Cell Dev Biol. 2021 Oct 14;9:740550. doi: 10.3389/fcell.2021.740550 (PMC8551707; doi:10.3389/fcell.2021.740550)
Supplement: Supplementary file 8 [file Data_Sheet_8.PDF]

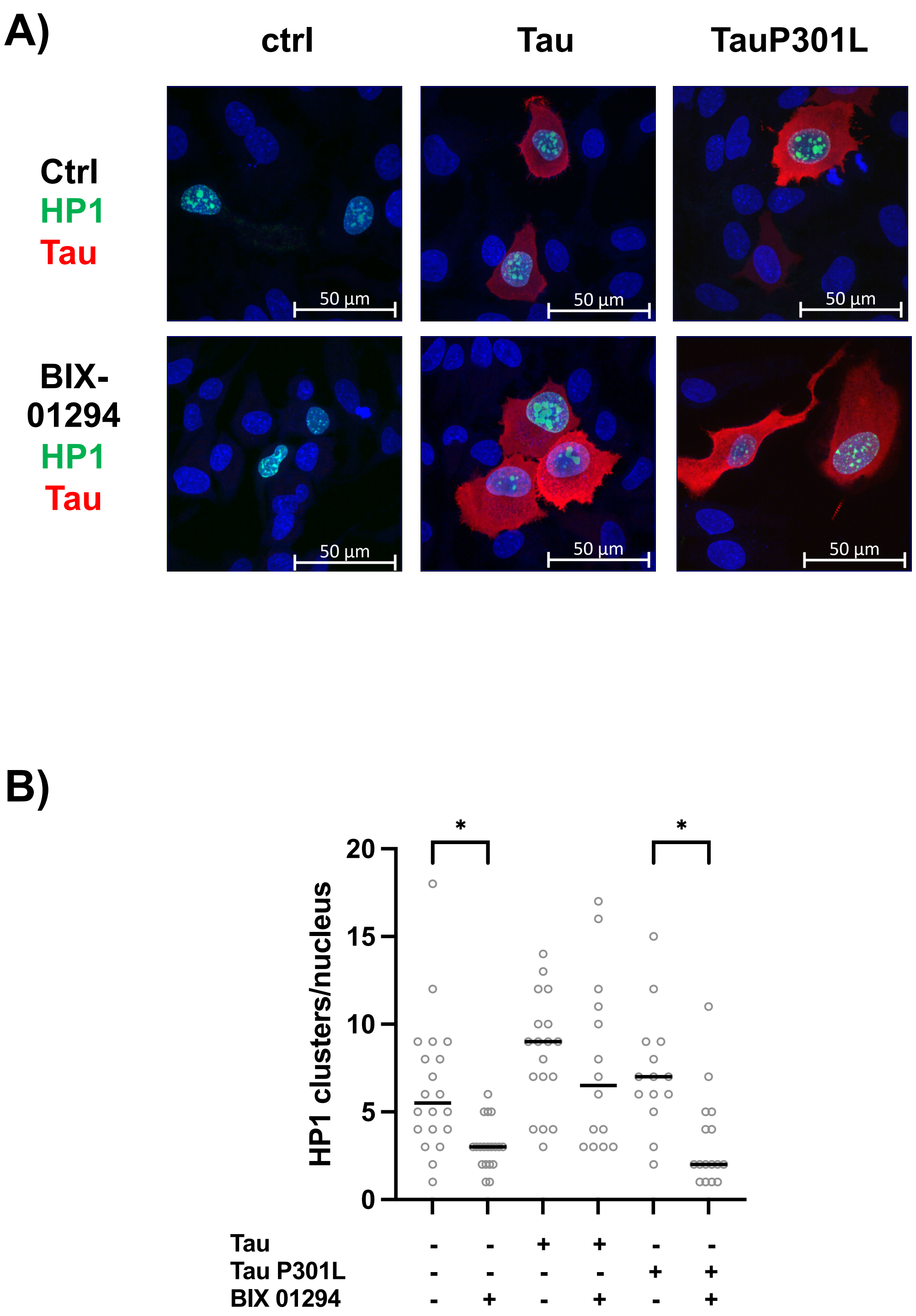

Supplementary Figure 8 : Effect of Tau4R or TauP301L on PCH structure under BIX 01294 treatment. (A) Single confocal sections of Hela cells transfected with GFP-HP1β, with or without Tau4R and treated 24h later BIX 01294 (6mM) for 24h. Tau C-ter antibodies and GFP fluorescence were used to visualize total Tau protein and HP1β respectively. Representative images are shown. (B) Quantification GFP-HP1β clusters *per* nucleus in Hela cells transfected with GFP-HP1β, with or without Tau4R or Tau4R P301L and treated 24h later BIX 01294 (6mM) for 24h. Tau C-ter antibodies and GFP fluorescence were used to visualize total Tau protein and HP1β respectively. Data are mean±SD. $^{*}P<0.05$.
