# Supplementary material for: Tau Stabilizes Chromatin Compaction
Source: Front Cell Dev Biol. 2021 Oct 14;9:740550. doi: 10.3389/fcell.2021.740550 (PMC8551707; doi:10.3389/fcell.2021.740550)
Supplement: Supplementary file 9 [file Data_Sheet_9.PDF]

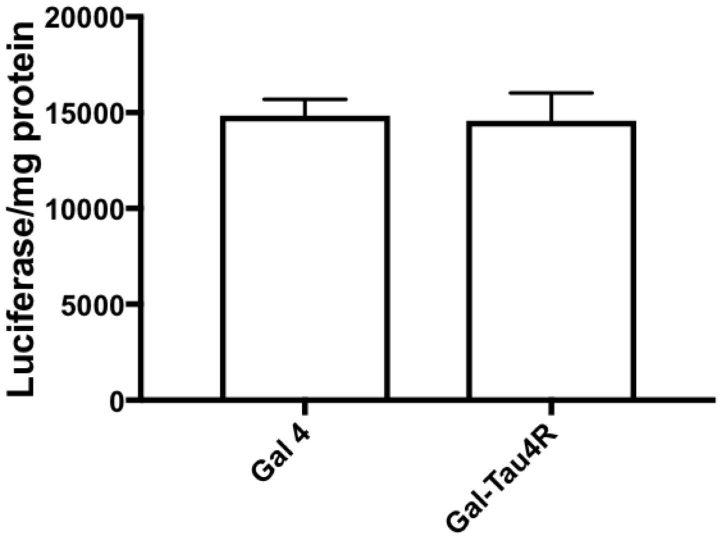

Supplementary Figure 9 : Absence of ectopic tethered Tau4R effects of on integrated reporter plasmid displaying high basal luciferase activity.

Tau tethering did not prevent adjacent integrated reporter plasmid activity when displaying high basal luciferase activity. pGL4.31[luc2P/GAL4 UAS/Hygro] reporter gene was stably transfected in Hela cells and selected for high basal luciferase activity. Stable clone was then transfected with Gal4DBD (Gal4) or Gal4DBD-Tau4R. The luciferase activity was determined as described in in the Materials and Methods. Data are mean±S.E.M.
