# Supplementary material for: Tau Stabilizes Chromatin Compaction
Source: Front Cell Dev Biol. 2021 Oct 14;9:740550. doi: 10.3389/fcell.2021.740550 (PMC8551707; doi:10.3389/fcell.2021.740550)
Supplement: Supplementary file 10 [file Data_Sheet_10.PDF]

Oligonucleotides	K_D (μM)
Seq 1 : ACATGTGGCCACAAATTGTTATCCGGTCACAATTGGACAT	2.09 ± 0.44
Seq 2 : CCTTCTCTTTATCATTCTCTCCCTCCTTCTTTCCCTCTCT	1.23 ± 0.4
Seq 3 : GAGGGAGAGAGAGGAAGACAGAGGAAGAGGAGGTGGAAG	0.8 ± 0.1
Seq 4 : ACAGGTGCAGAGGAAGATCTGGAGAGTGAGAGTGAGGCAG	2.16 ± 0.7
Seq 5 : CTCACTTTCCCCATTTCCACTCAGAACCCACTCCCCATCT	0.87 ± 0.23

Supplementary Table 1: Interaction between Tau4R and different oligonucleotides assessed by microscale thermophoresis. Oligonucleotide 1 (seq 1) was used as a control compared to different GAGA responsive elements

MST was conducted using a NT.115 Pico MST instrument (Nano Temper Technologies GmbH) equipped with red and blue filter sets. His-Tau protein, diluted to 200 nM in PBS-T buffer (supplied by vendor), was labeled with Monolith His-Tag Labeling Kit RED-tris-NTA (Nano Temper). The RED-tris-NTA dye was diluted in PBS-T to 100 nM. The mix was incubated at room temperature in the dark for 30 min. Oligonucleotides (50μM) were diluted with a serial 1:1 ratio of 16 gradients. Then the labeled protein and oligonucleotides were mix with 1:1 ratio and incubated at room temperature in the dark for 120 min. Capillaries are then filled individually and loaded into instrument. Data were acquired using high MST power and 100 % LED. Data were analyzed using MO Control Software (Nano Temper).
